# Systematic Review and Meta-Analysis of Correlates of FFMQ Mindfulness Facets

**DOI:** 10.3389/fpsyg.2019.02684

**Published:** 2019-12-06

**Authors:** Josef Mattes

**Affiliations:** Fakultät für Mathematik, Universität Wien, Vienna, Austria

**Keywords:** mindfulness, five-facet mindfulness questionnaire, FFMQ, meta-analysis, publication bias, non-judgment, moderators

## Abstract

**Background:** A number of meta-analyses of mindfulness have been performed, but few distinguished between different facets of mindfulness, despite it being known that facets of mindfulness behave differently in different populations; and most studied the outcome of interventions, which tend to involve additional ingredients besides mindfulness. Furthermore, there has recently been some concern regarding possible publication bias in mindfulness research.

**Objective:** Systematic review and meta-analysis of the relationship of different facets of mindfulness with various outcomes, taking into account possible moderators, and controlling for publication bias using a method appropriate given the substantial heterogeneity present.

**Methods:** Random effects meta-analysis with a number of robustness checks and estimation of the possible impact of publication bias on the results. Included are all studies that report correlations of outcomes with all five FFMQ facets, in English, French, German, or Spanish.

**Study Registration:** PROSPERO International prospective register of systematic reviews http://www.crd.york.ac.uk/PROSPERO/display_record.asp?ID=CRD42016041863.

**Results:** For the designated primary measure (SWLS) estimated correlations were: 0.15 [0.07, 0.22] for the Observing facet, 0.31 [0.27, 0.36] for Describing, 0.35 [0.31, 0.38] for Acting-with-Awareness, 0.30 [0.10, 0.47] for Non-judging and 0.28 [0.18, 0.37] for Non-reacting. Grouping all desirable outcomes together, Describing has the highest zero-order (though not partial) correlation; Non-judging the highest effect on avoiding undesirable outcomes. Results seem to be reasonably robust even to severe publication bias.

## Introduction

### Background

Work on mindfulness and its applications is booming, in considerable part due to the success of Mindfulness Based Stress Reduction (Kabat-Zinn, 2013). A number of meta-analyses confirm the usefulness of mindfulness in a range of applications from treatment of psychosis (Cramer et al., 2016), other psychological problems (Khoury et al., 2013), or symptoms of physical diseases (Rinske et al., 2015) to work life (Mesmer-Magnus et al., 2017) and sports performance (Bühlmayer et al., 2017), among others. Nevertheless, a number of concerns have been voiced in the literature relating to these results (e.g., Dam et al., 2018). The present paper will concentrate on two of these: possibly biased reporting of results and—more fundamentally—lack of clarity concerning the concept of mindfulness. The second issue will be discussed first.

Mindfulness seems to be conceptualized frequently in ways similar to Kabat-Zinn's (2003) operational working definition as being “the awareness that emerges through paying attention on purpose, in the present moment, and non-judgmentally to the unfolding of experience moment by moment.” Such an understanding of the construct has been criticized from a Buddhist point of view for being divorced from a traditional understanding of the term which was claimed to centrally involve remembrance of the Buddha's teachings (Levman, 2017), but this interpretation of the meaning of *sati* (mindfulness) in the Buddhist Pali canon is far from unanimous even among Buddhists (Anālayo, 2018), and there seems to be no clear reason why such remembrance should be relevant in a non-Buddhist context (Repetti, 2016; compare also Mattes, 2019). More importantly for present purposes, there is also disagreement about the mindfulness construct in the scientific literature, both concerning the nature and relative importance of its facets (with some contributions for example putting more stress on awareness and de-automatization, e.g., Kang et al., 2014), and even their number (questionnaire measures of mindfulness range from unidimensional ones like the Mindful Attention Awareness Scale, Brown and Ryan, 2003, to at least eight as in the Comprehensive Inventory of Mindfulness Experiences, Bergomi et al., 2015).

In this respect, the non-judging aspect of Kabat-Zinn's definition may deserve particular attention. It is easy to believe that being able to pay attention and to act on purpose are helpful: it may seem less clear that being in the present moment is necessarily beneficial (compare the idea of future-oriented prospection being central to human flourishing: Seligman et al., 2013), even less so why a non-judging attitude might be desirable. Indeed, other uses of the term “mindfulness” like Ellen Langer's (Ie et al., 2014) seem unconnected to the idea of non-judging of experience. Even more to the point, a non-evaluative stance toward one's experience (including thoughts and emotions) seems to be in conflict with the emphasis in cognitive therapy on challenging dysfunctional or irrational thoughts. For example, Rational Emotive Behavioral Therapy claims that

*you largely bring on your own emotional disturbances by choosing, both consciously and unconsciously, to think irrationally, to create unhealthy negative feelings, and to act in self-defeating ways […] therefore, you can choose to change your thinking, feelings and behaviors to undisturb yourself (Ellis, 1999, p. 175, emphasis in original)*.

Given the Buddhist roots of Kabat-Zinn's notion of mindfulness, it is also remarkable that most versions of traditional Buddhism do advise to develop *sammā-diṭṭhi* (usually translated “correct view”) and abandon *micchā-diṭṭhi* (“wrong view”), which seems difficult without evaluating one's thoughts.

Thus, there seems to be a need for a comprehensive study of the effects of different facets of mindfulness, both as a possible contribution to clear up conceptual confusions, and as an aid in designing effective and efficient interventions. Most existing meta-analyses do not study the differential effects of all mindfulness facets simultaneously. In addition, they tend to study (almost always relatively short-term) interventions, which has a number of drawbacks: first, mindfulness is usually considered to be trait-like, therefore slow to change, which makes short-term interventions a non-obvious setting for its study; second, interventions tend to have other ingredients (e.g., psycho-education and group effects in the case of MBSR) which might color the conclusions. Consistent with this, Eberth and Sedlmeier (2012) wrote in the conclusion of their meta-analysis of mindfulness meditation effects that they found large differences in effect sizes for MBSR vs. meditation and that “[t]his raises the question if some effect sizes found for MBSR might be partly inflated by effects that are not attributable to its mindfulness meditation component.” Also consistent with this, Rau and Williams (2016) argued for a distinction between dispositional and cultivated mindfulness. These considerations imply that, rather than studying only changes of outcomes during interventions, it may be a useful addition to the literature to study correlates of mindfulness facets cross-sectionally. Additional benefits of studying correlations will be discussed below.

As regards biased reporting, Coronado-Montoya et al. (2016) presented evidence for publication bias in the scientific literature on mindfulness. Among 124 published randomized controlled trials of mindfulness-based therapy in their survey, 108 (87%) reported positive outcomes; whereas the authors suggested that, based on power considerations, the expected proportion of positive outcomes would have been around 53%. In other words, the proportion of published non-positive results among all non-positive results (a measure of publication bias) may be little more than a quarter [as (1–87%)/(1–53%) = 0.28], indicative of considerable publication bias. In principle, there are a number of ways one can try to deal with publication bias. One, most easily used when dealing with a small number of studies, is to try to find and collect a sufficiently large proportion of those works that went unpublished. Alternatively, when a large number of studies is available, one can test for the presence of publication bias and/or try to estimate its possible impact on the results. In the present paper, this last course of action will be pursued for reasons explained below in the Methods section.

### The Present Study

Main aim of the present work is to contribute to the clarification of the relative importance of mindfulness facets for beneficial outcomes, while estimating the possible impact of publication bias.

In order to study facets of mindfulness in a large enough sample to control for publication bias, correlations with the most popular multi-facet self-report measure of mindfulness [the Five Facet Mindfulness Questionnaire (FFMQ) by Baer et al., (2006)], are meta-analized. The FFMQ was derived from a comprehensive study of self-report measures of mindfulness in use at that time. Psychometric analysis led to a five factor model, with facets labeled as: Observing, Describing, Acting-with-awareness, Non-judging of and Non-reacting to inner experience (these will be usually be abbreviated as Obs, Des, Act, NJ and NR, respectively, in the present paper). Observing refers to noticing and attending to sensations, perceptions, thoughts and feelings (example item: “I notice how foods and drinks affect my thoughts, bodily sensations, and emotions”), Describing means the propensity to label experience in words (e.g., “My natural tendency is to put my experiences into words”), the Acting-with-awareness subscale involves items like “I find myself doing things without paying attention,” Non-judging refers to the extent to which one judges one's own experiences (e.g., “I make judgments about whether my thoughts are good or bad”) and Non-reacting concerns one's tendency to immediately react to situations, feelings or thoughts (e.g., “When I have distressing thoughts or images, I just notice them and let them go”). The observe facet is known to behave differently among participants that meditate vs. those that do not (Baer et al., 2008) which suggests that moderators of the correlations of mindfulness facets with outcomes are also an interesting topic to study.

For the above reasons, the present study performs a meta-analysis of correlates of facets of mindfulness as measured by the FFMQ. Besides being interesting in itself, studying correlations has the additional benefit of leading to a large sample size, which—as mentioned above—is important for the chosen method to study the impact of possible publication bias. Given another important recent concern, namely research standards in psychology (e.g., Bones, 2012; Brown et al., 2013; Open Science Collaboration, 2015; Ledgerwood, 2016), another aspect that is taken into account is adherence to (two aspects of) good research practice (Finkel et al., 2015): preregistration (Moore, 2016) and transparency; the latter will be proxied by availability (whether the underlying data used and/or the paper are openly available) and readability (whether the abstract is structured or not). Partial correlations with outcomes will also be studied, as the unique contributions of facets seem interesting both in themselves (above it was argued that for some facets positive effects might seem counter-intuitive, thus it seems important to exhibit to what extent they uniquely contribute to positive outcomes) and in the design of mindfulness trainings and interventions (by indicating which facets should be preferentially strengthened).

One aspect of the existing studies of mindfulness that Coronado-Montoya et al. (2016) criticized was, that few of them had designated a primary outcome measure, which can lead to biased presentation of results through overemphasis on positive outcomes. For the present paper, two primary outcome measures (satisfaction with life and purpose in life) were chosen from positive psychology (Seligman and Csikszentmihalyi, 2000), for the following reasons: On the one hand, extant mindfulness research seems to primarily concentrate on therapy rather than on furthering happiness or flourishing (e.g., a quick search in *pubpsych* for “mindfulness” + “therapy” found 2,378 results, compared to 51 for “mindfulness” + “happiness” and 20 for “mindfulness” + “flourishing”). On the other hand, arguably the aim of mindfulness practice is not only therapeutic, but to contribute to “flourishing on this planet […] for the benefit of all sentient beings and our world” (Kabat-Zinn, 2011). Nor need the Buddhist roots of mindfulness force us into a mindset overemphasizing curing the negative, as “Buddhist methods bring courage, joy, power, and the richness of love” (Nydahl, 2008) rather than just relief from suffering; originally, Buddhism may have put a lot less emphasis on the latter as is commonly perceived (Anālayo, 2017; Mattes, under review). As a contribution to redress this imbalance, the primary emphasis in this study will be on positive psychology measures. Given that there is a widely assumed distinction between well-being and eudaimonia—with meaning in life being a particularly distinctive feature of eudaimonia (Disabato et al., 2016)—at the preregistration stage two measures were designated as primary outcomes for this meta-analysis: the Satisfaction With Life Scale (SWLS, e.g., Diener et al., 1985; Pavot and Diener, 2008) as a widely used measure of well-being, and the Purpose in Life Scale (PiL) as the most widely used measure of meaning (Bronk, 2014). Secondary outcomes were all other measures, individually if at least four data points are available for meta-analysis, otherwise grouped as described in the methods section below.

## Methods

### Study Registration

This study was registered with the PROSPERO International prospective register of systematic reviews (Booth et al., 2012): http://www.crd.york.ac.uk/PROSPERO/display_record.asp?ID=CRD42016041863, in accordance with the Preferred Reporting Items for Systematic reviews and Meta-Analyses (PRISMA, Moher et al., 2009, 2015).

### Database Search

On July 15, 2016, the following files were downloaded from the website of the American Mindfulness Research Association (https://goamra.org/resources/mindfo-database/): AMRA_database_1966-2015.zip, Jun2016_AMRA_library.zip, AMRA_database_feb2016.zip, Mar2016_AMRA_database.zip, Apr2016_AMRA_database.zip, May2016_AMRA_library.zip, Jan2016_AMRA_database-.zip. On the same date, the following databases were searched for the terms “FFMQ” and “Five facet^*^ mindfulness”: PubMed, PsycINFO, ResearchGate. The search resulted in 1,229 potentially relevant papers, 708 after removing duplicates.

### Study Selection

Included in the meta-analysis were all studies which (a) reported correlations with all five mindfulness facets for any outcome that was not another mindfulness measure, where (b) both the publication and the questionnaire used were in one of the following languages: English, French, German, Spanish. Excluded were studies which reported only changes (not levels) of mindfulness facets, and those using a short form of the FFMQ (Park et al., 2013, in their survey of mindfulness measures, also excluded shortened or modified versions).

A controversial issue in meta-analysis is whether unpublished “gray” literature should be included. Even though most authors seem to advocate including as many studies as possible, there are good reasons against it (e.g., Ferguson and Brannick, 2012), in particular if few studies are pre-registered (as is the case here) and if the number of published effect sizes is large (again the case here) so that one can use statistical techniques (as for example discussed in McShane et al., 2016 or in Jin et al., 2015) to adjust for publication bias. Also, according to the comprehensive study of bias in science performed by Fanelli et al. (2017), studies not published in peer-reviewed journals tend to underestimate effects. For these reasons, the present meta-analysis includes only published studies.

Papers found during database search were checked for relevance by scanning the PDF of the article where available, else abstracts were examined whether they mentioned FFMQ facets. This resulted in four requests to authors to send their paper, one of which was fulfilled. For book chapters, Google books was searched if neither the book nor a PDF were available.

**Figure 1:**
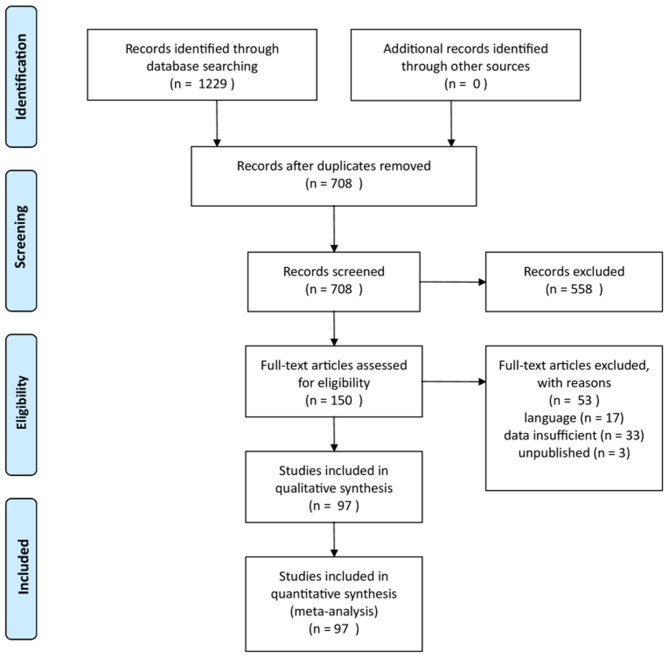
PRISMA flow diagram.

This procedure resulted in a final sample of 117 studies in 97 publications (see Figure 1 and Supplementary Tables 1, 2).

### Data Extraction

From each paper the following data were collected: publication type, authors, year of publication, number of relevant studies, number of samples used, number of measures used; whether the paper was open access or available via ResearchGate or PubMed, whether a link was given to access the underlying data (this was never the case), whether the abstract was structured, and whether the study reported was pre-registered.

For each sample, the following were collected, where available: number of participants, whether the sample was clinical, non-clinical or mixed, whether the participants were meditators, non-meditators or mixed, the participants' occupation (students; academics; other university community—e.g., administrators; health professionals; other professionals; military; community sample; internet sample), proportion of males in the sample (between 0 and 1), mean age, standard deviation of age, world region, and country in which the study was conducted, correlation table of the FFMQ scales, means and standard deviations and Cronbach alpha of the FFMQ scales, whether alpha was given precisely or as a range or quoted from another paper, correlation table of the outcome measures, primary outcome measure (in a few cases measures were assumed to be primary, for example based on the title of the paper, or giving preference to full scales relative to sub-scales; see also the Discussion below).

For each outcome measure, the correlations with the FFMQ facets together with the type of measure and directionality (see below) were collected. In addition, the following were collected, where available: Mean and standard deviation and Cronbach alpha. As a partial test of data integrity, a simple version of the GRIM test (Brown and Heathers, 2017) was performed: no irregularities were found. This selection procedure resulted in 565 effect sizes (correlations) per facet.

Effect sizes were grouped as follows: (1) Well-being/happiness (e.g., the Satisfaction With Life Scale: SWLS), (2) Eudaimonia (e.g., Psychological Well-Being: PWB), (3) Clinical/health-related (e.g., Depression Anxiety Stress Scales: DASS), (4) Physiology/body/movement (e.g., 6 Min Walk test), (5) Judgement and rationality (e.g., Monetary Choice Questionnaire), (6) Social behavior (e.g., relations subscale of PWB), (7) unhelpful (outcomes that might not be in themselves pathological but conceivably might have negative consequences, e.g., DERS = Difficulties in Emotion Regulation Scale), (8) helpful (outcomes that might not be in themselves desirable but may well have positive consequences, e.g., Health-Promoting Lifestyle Profile), (9) other. This last group of effect sizes was excluded from analysis, since aggregating correlations requires directionality in the outcomes (a higher score must be more desirable), which the measures in this group do not satisfy; for the same reason, signs of groups 3 and 8 had to be reversed to make directionalities consistent, as well as in a few individual studies where reversed scores were reported (e.g., Curtiss and Klemanski, 2014 for the AAQ-II scale). In addition, the body mass index, though obviously belonging in group 4, was excluded in non-overweight samples, because of lack of clear directionality (“less is better” is far from clear in non-clinical samples: being severely underweight is no more desirable than being severely overweight—just think of anorexia).

The final sample contained 528 effect sizes per FFMQ facet, plus 309 effect sizes for the FFMQ total score, altogether 2,859 usable effect sizes. There were three unusually large samples among the studies included in this meta-analysis: Camilleri et al. (2015) had two samples with 49,228 and 14,400 participants, respectively, Jones et al. (2015) provided one sample with 4,986 participants. Excluding those three, sample sizes ranged from 20 to 1,210 with mean 249.3 and median 179.

### Software

Calculations were performed using the free software environment for statistical computing and graphics R (R Core Team, 2012), version 3.4.0 under Windows 10, employing the packages “Matrix,” “weightr,” “meta,” and “corpcor,” (see also Schwarzer et al., 2015).

### Risk of Bias, Quality Assessment

Since this meta-analysis covers only correlational studies, no risk of bias assessment at the individual study level was performed, because the usual measures of bias risk (e.g., form of randomization) are geared toward intervention studies and for the most part cannot be applied here.

Concerning publication bias, it was originally intended to test for its presence using funnel plots and trim-and-fill as is common in meta-analyses of mindfulness. Nevertheless, these methods are not well-behaved under heterogeneity (Terrin et al., 2003). In view of the fact that heterogeneity in the present dataset turned out to be unexpectedly large in most cases (see below), and noting that the recently introduced techniques of p-curve and p-uniform also seem problematic already under moderate heterogeneity (van Aert et al., 2016; Carter et al., 2019), possible consequences of publication bias are assessed in this study using the three-variable selection method of Vevea and Hedges (1995) and Vevea and Woods (2005), as implemented in Coburn and Vevea (2016): effect estimates are reported under different probabilities for a statistically insignificant (two-sided *p*-value less than 5%) result getting published. This allows quantification of the possible influence of publication bias on mindfulness research, which (in the context of intervention studies) was noted in Coronado-Montoya et al. (2016).

Quality of studies was assessed in terms of transparency: low, medium (paper is published open access, or a version of the paper can be downloaded, e.g., from ResearchGate), high (underlying data available for download). In addition, the quality of the abstract was assessed by considering a structured abstract as higher quality (more user friendly, since it makes it easier to grasp crucial information quickly).

### Data Synthesis

#### Planned Studies

Pre-registration stipulated random effects meta-analysis, with tau-squared and *I*-squared as measures of heterogeneity (with *I*^2^ < 0.3 considered mild and *I*^2^>0.5 indicating severe heterogeneity, following Higgins and Thompson, 2002), for the following outcomes: SWLS (Satisfaction With Life Scale) as planned; in addition all measures (excluding sub-scales) with at least four data points, these were the following: PWB (Psychological Well-Being), PSWQ (Penn State Worry Questionnaire), DASS (Depression Anxiety Stress Scales), BDI_II (Beck Depression Inventory), PANAS_NA (Positive And Negative Affect Schedule—negative affect), PSS (Perceived Stress Scale), AAQ_II (Acceptance and Action Questionnaire).

Where possible, separate subgroup analyses were performed for groups described as: clinical vs. non-clinical vs. mixed, meditators vs. non-meditators vs. mixed, and for self-report vs. objective measures; in addition, meta-regressions were performed with publication year as the independent variable. The possible impact of publication bias was assessed as explained above.

Almost all studies measured several outcomes for each participant, but hardly any designated primary outcome measures, and only 41 of 117 studies reported covariances between the different outcomes or the data needed to calculate these, which made it impossible to properly aggregate the effect sizes. This problem was dealt with in the following way: First, all outcomes were included where appropriate, implicitly making the (unrealistic) assumption of zero correlation between the measures; as a robustness check, for each such case of multiple measurement, one measure was selected for inclusion (randomly, subject to the following constraints: full scales were preferred to subscales, and preference was given to types of outcomes as follows: well-being > eudaimonia > clinical > helpful > unhelpful > the remaining outcome types).

#### Additional Studies

In addition to the planned studies, efforts were made to explore the reasons for the surprisingly high heterogeneity in effect sizes, which was present even where single (rather than grouped) outcome measures were used. Specifically, where individual studies seemed to be outliers the meta-analysis was rerun with these studies removed. In addition, subgroup analyses based on region (in which part of the world the study was conducted) and professional background of the participants (students, community samples, academics, etc.) and meta-regressions based on mean age, standard deviation of age, and shares of males among the participants were conducted; also the correlations of outcomes with differences of facets were calculated and meta-analyses performed on these (results are available from the author).

## Results

Note: Given the need to adjust for multiple testing, *p*-values are reported to four digits throughout this paper. Effects will be reported to two digits to make reading large tables easier.

### Primary Outcome: Satisfaction With Life Scale (SWLS)

Designated primary outcomes for this meta-analysis were the Satisfaction With Life Scale (Diener et al., 1985), a widely used measure of well-being, and the Purpose in Life questionnaire. Despite the Purpose in Life questionnaire being the most used measure of meaning in life, not a single study included in the present sample used it; in fact, no measure of meaning was. Hence, SWLS is the sole primary outcome used in this meta-analysis.

**Figure 2:**
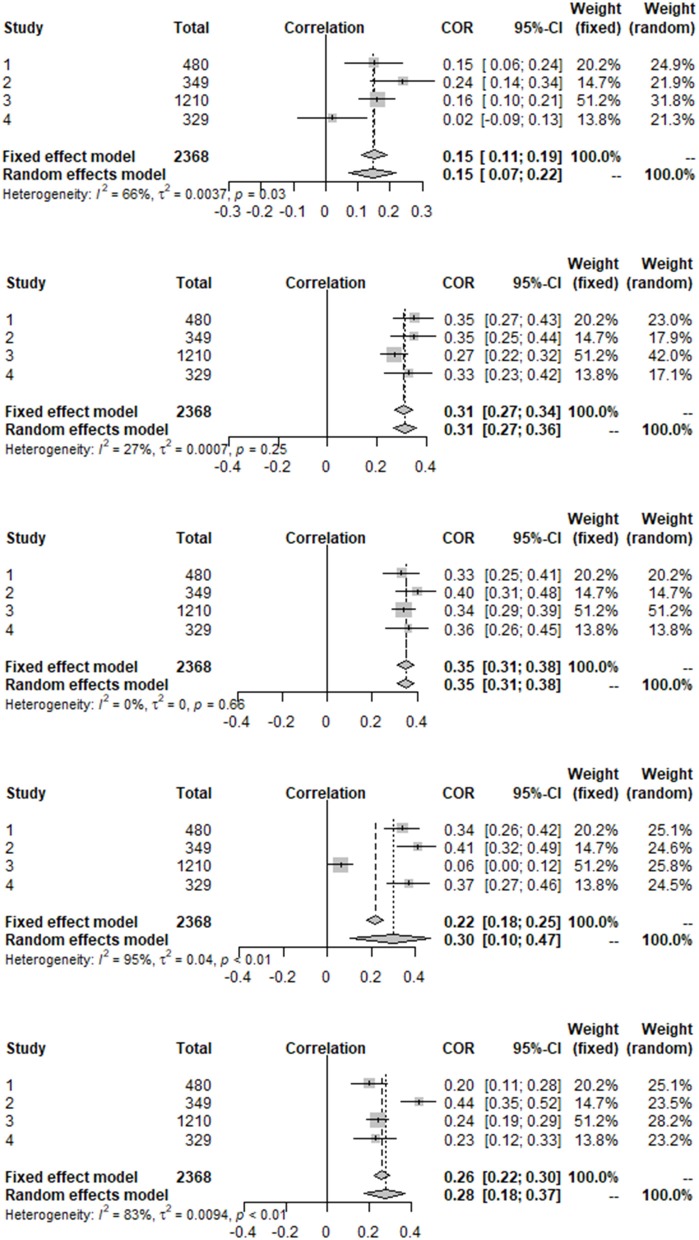
Forest plots for SWLS.

Four studies used the Satisfaction With Life Scale, for three of them it was possible to calculate partial correlations. Aggregate results from the meta-analyses both for the full sample, as well as restricted to those studies with partial correlations available, are presented in Table 1, the forest plots for the full sample are shown in Figure 2.

**Table 1:** Meta-analysis for SWLS.

**Facet**	**All studies**								**Partial corr. available**			
	***r***	**Lower**	**Upper**	***p***	***Q***	***Q*.pval**	***I_sq***	***tau_sq***	***r***	***p***	***r_p***	***p_p***
Obs	0.15	0.07	0.22	0.0001	8.76	0.0326	0.66	0.00	0.17	<0.0001	0.08	0.4877
Des	0.31	0.27	0.36	<0.0001	4.09	0.2516	0.27	0.00	0.31	<0.0001	0.16	0.1220
Act	0.35	0.31	0.38	<0.0001	1.61	0.6562	0.00	0.00	0.35	<0.0001	0.14	0.0060
NJ	0.30	0.10	0.47	0.0028	64.78	<0.0001	0.95	0.04	0.27	0.0259	0.20	0.0435
NR	0.28	0.18	0.37	<0.0001	17.49	0.0006	0.83	0.01	0.29	<0.0001	0.11	0.0774

*r, estimated correlation from random effects model; lower and upper, boundaries of the 95% confidence interval; p, p-value of r = 0; I_sq, I^2^ measure of heterogeneity; tau_sq, τ^2^ measure of heterogeneity; Q, measure of heterogeneity and Q.pval, p-value of Q = 0; r_p, partial correlation; p_p, p-value of r_p = 0*.

For both the full and restricted sample, Acting-with-awareness had the highest correlation with life satisfaction (the estimates for the other four facets are outside the 95% confidence interval for Act), with Describing, Non-judging and Non-reacting estimated to be close to each other, and Observing having a considerably weaker correlation (estimates for the other facets being far away from the confidence interval for Obs). The latter is unsurprising since none of the samples consisted of meditators (though one was mixed and did indeed reported the highest correlation for Obs, which nevertheless at 0.24 was still lower than the other estimated effects).

Table 1 revealed considerable heterogeneity and wide confidence intervals for NR and in particular for NJ. Inspection of the individual studies showed that the heterogeneity in NJ stemmed from the exceptionally small effect size reported in Lara et al. (2015). Indeed, eliminating that paper from the sample reduces *I*^2^ for both NJ and Des to zero (but it increases the already substantial heterogeneity for NR to 0.88). Unsurprisingly, the estimated correlation of NJ is increased (to 0.37), the other correlations are then estimated to be 0.36 (Act), 0.34 (Des), 0.29 (NR), and 0.14 (Obs).

The number of studies was too small to estimate the possible impact of publication bias or for subgroup comparisons. Meta-regressions found little evidence against the null hypothesis of independence from the regressor variables (of 20 *p*-values, only three were smaller than 0.05; none was smaller than the value of 0.0025 which results from Bonferroni-adjustment for 20-fold testing).

### Individual Secondary Outcomes

**Figure 3:**
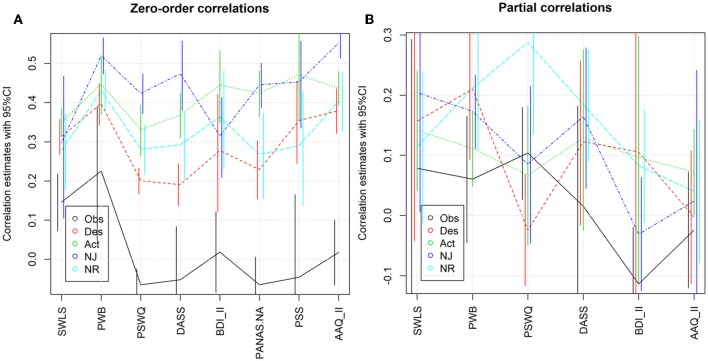
Individual outcomes. **(A)** Correlations and 95% confidence intervals. **(B)** Partial correlations and 95% confidence intervals.

This section reports the results for all measures for which at least four effect sizes were available, except SWLS (reported above) and subscales of any measure that is reported here (these concerns the three sub-scales of DASS), resulting in the following collection of outcomes: PWB (*k* = 6 effect sizes with *N* = 974 participants), PSWQ (*k* = 7, *N* = 3,302), DASS (*k* = 6, *N* = 1,252), BDI II (*k* = 4, *N* = 519), PANAS.NA (*k* = 4, *N* = 773), PSS (*k* = 4, *N* = 1464), AAQ II (*k* = 4, *N* = 1319). Note that all measures are used in such a way that higher values represent more desirable outcomes, so that all except PWB are inverted from their usual direction. Table 2 together with Figure 3 summarize the results.

**Table 2:** Meta-analyses for individual outcomes.

	**Correlations**					***p*****-values**				
**Outcome**	**Obs**	**Des**	**Act**	**NJ**	**NR**	**Obs**	**Des**	**Act**	**NJ**	**NR**
SWLS	0.15	0.31	0.35	0.30	0.28	0.0001	<0.0001	<0.0001	0.0028	<0.0001
PWB	0.23	0.40	0.45	0.52	0.43	0.0135	<0.0001	<0.0001	<0.0001	<0.0001
PSWQ	−0.07	0.20	0.33	0.42	0.28	0.0017	<0.0001	<0.0001	<0.0001	<0.0001
DASS	−0.05	0.19	0.37	0.47	0.29	0.4502	<0.0001	<0.0001	<0.0001	<0.0001
BDI_II	0.02	0.28	0.44	0.31	0.36	0.7260	0.0006	<0.0001	<0.0001	<0.0001
PANAS.NA	−0.07	0.23	0.42	0.45	0.27	0.0723	<0.0001	<0.0001	<0.0001	<0.0001
PSS	−0.05	0.35	0.47	0.45	0.29	0.6739	<0.0001	<0.0001	<0.0001	0.0002
AAQ_II	0.02	0.38	0.44	0.55	0.40	0.6911	<0.0001	<0.0001	<0.0001	<0.0001

*SWLS, Satisfaction With Life Scale; PWB, Psychological Well-Being; PSWQ, Penn State Worry Questionnaire; DASS, Depression and Anxiety Scales; BDI_II, Beck Depression Inventory II; PANAS.NA, negative affect subscale of PANAS; PSS, Perceived Stress Scale; AAQ_II, Acceptance and Action Questionnaire II*.

The Observing facet showed small effect sizes for all well-being and eudaimonic outcomes, and zero-to-negative effects for the other categories (the correlation with PSWQ in particular was statistically significantly negative with *r* = −0.07 and *p* = 0.0017). In any case, the effects were much smaller than those of the other facets—in almost all cases the 95% confidence intervals were disjoint. Overall, there seemed to be a weak tendency for NJ to have the largest effect, followed by Act, NR and then Des, but confirmation or dis-confirmation of this requires more repeated use of these measures to arrive at more precise estimates. The last statement applies even more clearly for partial correlations, for which the estimates are too imprecise to draw conclusions. In addition to the data reported in the table, heterogeneity was high in many cases, with 21 of 40 *I*^2^ statistics being above 0.5.

For PWB, correlations were estimated to be large for NJ [*r* = 0.52, 95% confidence interval = [0.4727, 0.5651)], small for Obs [*r* = 0.23, CI = (0.0475, 0.3902)], and medium (between 0.45 and 0.4) for the other three facets. All estimates except that for Obs were highly robust to assumptions about publication bias. Heterogeneity was a substantial concern for Obs (*I*^2^ = 0.87), borderline severe for Act (*I*^2^ = 0.52), and negligible for the other three facets. For Observing it is noticeable that three studies reported medium effects (*r* between 0.3 and 0.45), whereas the other three studies reported very small effects (between −0.02 and 0.08). Interestingly, this is not entirely due to the difference between meditators and non-meditators: the samples reporting medium effects were one meditator, one mixed and one non-meditator sample (the other three samples consisted of non-meditators). Concerning possible moderators, meta-regressions found again no evidence against the null hypothesis of independence from the regressor variables (all *p* ≥ 0.1), while the number of studies was too small for subgroup comparisons.

As noted above, PSWQ showed a small but statistically significant (*p* = 0.0017) negative effect of Obs, and highly significant (all *p* < 0.0001) small-to-medium effects of the other facets. Heterogeneity was elevated but not extreme for Act, NJ and NR. Again, meta-regressions found little evidence against the null hypothesis of independence from the regressor variables (only four *p*-values were smaller than 0.05; none was smaller than the Bonferroni-adjusted value of 0.0025). The number of studies was too small for subgroup comparisons, nor could the model for publication bias be estimated.

Results for DASS were similar to those for PSWQ, except that the negative effect of Obs was statistically insignificant and the effect of NJ was increased slightly. Furthermore, for DASS the model for publication bias could be estimated: Even with the assumption of very severe publication bias (90% of statistically insignificant results going unpublished), the estimated correlations are only slightly reduced to −0.1073 for Obs, 0.1609 for Des, 0.3494, for Act, 0.4369 for NJ, and 0.2583 for NR.

The pattern of results is generally similar for BDI II and PANAS.NA. For the PSS it is interesting to note that Obs shows correlations with the outcome measure ranging from −0.21 to +0.16, with both of the extreme values estimated in non-meditating student samples in the USA. Finally, AAQ II is the only outcome measure besides PWB where a large correlation was estimated (for NJ, *r* = 0.55).

### Grouped Outcomes: Overview

The grouping of measures that were not used often enough to allow individual meta-analysis has a substantial subjective component. Probably the most subjective choice is that of assigning desirable outcomes to well-being, eudaimonia, or what is here called helpful, on the one hand (including the individually reported SWLS and PWB), and of undesirable ones to clinical vs. unhelpful on the other (again including the individually reported measures). Therefore, the presentation will focus on results for desirable outcomes grouped together, and undesirable outcomes grouped together, plus a few remarks on the results related to body or social outcomes (which are based on much smaller samples). Results for subsets of measures (e.g., Other_Eudaimonia defined as eudaimonia excluding PWB) can serve as a robustness check.

**Figure 4:**
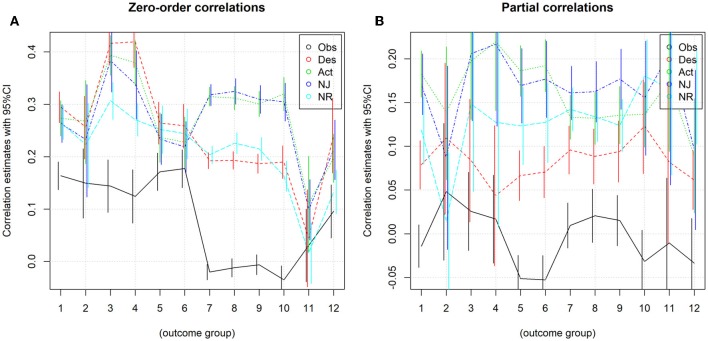
Grouped outcomes. **(A)** Correlations and 95% confidence intervals. **(B)** Partial correlations and 95% confidence intervals.

The grouped results seem to confirm the results for corresponding individual measures as Obs has only small correlations with positive outcomes, and no effect at all on undesirable outcomes. Most other correlations are highly statistically significant and of practically meaningful magnitude, even unique contributions assessed by partial correlations range up to 0.22 with most *p* < 0.0001 (see Table 3 and Figure 4).

**Table 3:** Partial correlations, correlations, and corresponding p-values for grouped outcomes.

**Group**	**#**	**Partial correlations**					**Correlations**				
		**Obs**	**Des**	**Act**	**NJ**	**NR**	**Obs**	**Des**	**Act**	**NJ**	**NR**
All_desirable	1	−0.01 0.2545	0.08 <0.0001	0.18 <0.0001	0.17 <0.0001	0.12 <0.0001	0.17 <0.0001	0.31 <0.0001	0.29 <0.0001	0.29 <0.0001	0.29 <0.0001
Other_WB&H	2	0.05 0.2290	0.11 0.0138	0.14 0.0004	0.09 0.1039	0.01 0.7206	0.16 <0.0001	0.25 <0.0001	0.24 <0.0001	0.23 <0.0001	0.21 <0.0001
Other_eudaimonia	3	0.02 0.5121	0.04 0.2849	0.22 <0.0001	0.22 <0.0001	0.13 0.0375	0.12 <0.0001	0.42 <0.0001	0.38 <0.0001	0.34 <0.0001	0.27 <0.0001
helpful	4	−0.05 0.0002	0.07 <0.0001	0.19 <0.0001	0.17 <0.0001	0.12 <0.0001	0.18 <0.0001	0.27 <0.0001	0.25 <0.0001	0.26 <0.0001	0.28 <0.0001
Other_helpful	5	−0.05 0.0002	0.07 <0.0001	0.19 <0.0001	0.18 <0.0001	0.13 <0.0001	0.18 <0.0001	0.26 <0.0001	0.24 <0.0001	0.24 <0.0001	0.27 <0.0001
All_undesirable	6	0.01 0.4713	0.10 <0.0001	0.13 <0.0001	0.16 <0.0001	0.14 <0.0001	0.00 0.8852	0.20 <0.0001	0.32 <0.0001	0.34 <0.0001	0.23 <0.0001
Clinical	7	0.02 0.1858	0.09 <0.0001	0.13 <0.0001	0.16 <0.0001	0.13 <0.0001	−0.01 0.6647	0.20 <0.0001	0.33 <0.0001	0.34 <0.0001	0.24 <0.0001
Clinical_no_indiv	8	0.02 0.3026	0.09 <0.0001	0.14 <0.0001	0.18 <0.0001	0.12 <0.0001	0.00 0.9081	0.19 <0.0001	0.31 <0.0001	0.33 <0.0001	0.22 <0.0001
unhelpful	9	−0.03 0.0832	0.12 <0.0001	0.14 <0.0001	0.16 <0.0001	0.18 <0.0001	0.03 0.4353	0.20 <0.0001	0.31 <0.0001	0.33 <0.0001	0.19 <0.0001
Body	10	−0.01 0.7806	0.08 0.0852	0.18 <0.0001	0.21 0.0076	0.17 0.0010	0.02 0.5877	0.02 0.6743	0.14 0.0057	0.10 0.0014	0.02 0.5761
Social	11	−0.03 0.1976	0.06 0.0004	0.09 0.0077	0.10 0.0397	0.16 <0.0001	0.13 0.0002	0.25 <0.0001	0.19 <0.0001	0.21 <0.0001	0.16 <0.0001

### Desirable Outcomes

Combining the groups “well-being,” “eudaimonia,” “helpful” (including SWLS and PWB) led to 328 effect sizes and the following aggregate results:

Obs showed a small effect (*r* = 0.16), the other four facets medium effects (between 0.26 and 0.29) with confidence intervals disjoint from that for Obs. Nevertheless, heterogeneity is a substantial concern, given that all *I*^2^ are greater than 0.85. Table 4 suggests that the effect of Des is slightly larger than that of Act, NJ and NR; meta-analysis of the effect of differences of facets (where these could be calculated) showed that this is indeed statistically significant (*p* = 0.0219).

**Table 4:** Meta-analysis for desirable outcomes.

**Facet**	**All**								**w/p**			
	***r***	**Lower**	**Upper**	***p***	***Q***	***Q*.pval**	***I*_sq**	**tau_sq**	***r***	***p***	***r*(pc)**	***p*(pc)**
Obs	0.16	0.14	0.19	<0.0001	1,014.21	<0.0001	0.86	0.02	0.17	<0.0001	−0.01	0.2545
Des	0.29	0.26	0.32	<0.0001	1,470.72	<0.0001	0.90	0.03	0.31	<0.0001	0.08	<0.0001
Act	0.27	0.24	0.31	<0.0001	1,886.43	<0.0001	0.92	0.04	0.29	<0.0001	0.18	<0.0001
NJ	0.26	0.23	0.30	<0.0001	2,172.98	<0.0001	0.93	0.05	0.29	<0.0001	0.17	<0.0001
NR	0.27	0.23	0.30	<0.0001	1,701.14	<0.0001	0.92	0.04	0.29	<0.0001	0.12	<0.0001

*r, estimated correlation from random effects model; lower and upper, boundaries of the 95% confidence interval; p, p-value of r = 0; I_sq, I^2^ measure of heterogeneity; tau_sq, τ^2^ measure of heterogeneity; Q, measure of heterogeneity; Q.pval: p-value of Q = 0; r(pc), partial correlation; p(pc), p-value of r(pc) = 0. All = all studies, w/p: those with partial correlations available*.

Interestingly, partial correlations revealed that the unique contribution of Des is much smaller than of Act and NJ [again with disjoint confidence intervals: (0.05, 0.11) for Des vs. (0.16, 0.21) for Act and (0.14, 0.21) for NJ].

Results seem robust to reasonable levels of publication bias: If 90% of studies with a statistically insignificant result go unpublished, the estimated effects would still be at least 0.19 for all facets except Obs (Table 5).

**Table 5:** Robustness to publication bias for desirable outcomes.

	**1-sided**					**2-sided**				
**Prob**	**Obs**	**Des**	**Act**	**NJ**	**NR**	**Obs**	**Des**	**Act**	**NJ**	**NR**
1	0.16	0.29	0.27	0.26	0.26	0.16	0.29	0.27	0.26	0.26
0.7	0.16	0.28	0.26	0.25	0.25	0.15	0.27	0.25	0.24	0.24
0.4	0.15	0.28	0.25	0.23	0.24	0.13	0.25	0.23	0.22	0.22
0.2	0.14	0.28	0.24	0.22	0.22	0.11	0.23	0.21	0.20	0.20
0.1	0.13	0.27	0.24	0.20	0.21	0.10	0.22	0.20	0.19	0.19

*Prob, probability that a result with p-value less than 0.05 gets published*.

Regressions of effect sizes on possible moderators show some evidence of moderating influences by the tested variables: Even after Bonferroni-correction for 20-fold testing, the effect of NJ seems to decrease for later publications. NR might also be negatively related to publication year, though the *p*-value is slightly less convincing, all other coefficients for publication year are also negative, but with *p*-values far above thresholds for statistical significance. Similarly, the coefficients for mean age and proportion of males are all negative, with the following being significant or close to significant after Bonferroni correction: the effect of Des decreases with mean age of the sample, that of Obs and NR seems to decrease with the proportion of males among the participants. Overall, it appears difficult to discern a pattern in these moderator results.

Table 6 presents the results for clinical vs. non-clinical participants, and for meditators vs. non-meditators. Among 27 non-clinical compared to seven clinical samples (providing 125 and 17 effect sizes, respectively) all facets show a substantially lower effect in clinical samples, with a *p* < 0.001 for Observing and Describing, and *p* < 0.1 for the other facets. Eighteen samples consisted of non-meditators, three of meditators, and 17 were mixed. For all facets the highest effect was estimated in the mixed groups, this being statistically significant at the 0.05 level for four of the five facets, and for two this still holds after correction for multiple testing. Nevertheless, as a reviewer pointed out, this may be a statistical artifact.

**Table 6:** Desirable outcomes: clinical vs. non-clinical and meditator vs. non-meditator samples.

	**Obs**	**Des**	**Act**	**NJ**	**NR**	***n*(e)**	***k***	***N***
Non-clinical	0.18	0.31	0.28	0.27	0.27	125	27	6,831
Mixed	N/A	N/A	N/A	N/A	N/A			
Clinical	0.03	0.15	0.16	0.14	0.16	17	7	2,010
p(equal)	0.0001	0.0006	0.0739	0.0775	0.0952			
Non-meditators	0.13	0.28	0.27	0.24	0.24	107	18	5,613
Mixed	0.26	0.37	0.32	0.35	0.37	28	17	2,867
Meditators	0.26	0.16	0.2	0.24	0.25	9	3	1,252
p(equal)	0.0004	0.0182	0.1675	0.0130	0.0001			

*Within-subgroup estimates of effect sizes, and p-values from F-test for equality of effects across subgroups. n(e), number of effect sizes; k, number of studies; N, number of participants*.

There is also strong statistical evidence for dependence of effects on occupation, in particular, NJ and NR showed the largest effects in the samples consisting of academics, and very small effects among the (few) samples consisting of professionals, as Table 7 reveals:

**Table 7:** Desirable outcomes by occupation.

	**Obs**	**Des**	**Act**	**NJ**	**NR**	***n*(e)**	***k***	***N***
Academic	0.3	0.32	0.31	0.47	0.44	9	6	409
Students	0.14	0.33	0.3	0.28	0.26	83	23	6,502
Community	0.13	0.21	0.23	0.2	0.25	21	8	2,043
Professional	0.2	0.2	0.25	0.04	0.07	8	3	744
Internet	0.32	0.37	0.29	0.27	0.37	10	6	2,010
Health	0.16	0.04	0.11	0.15	0.24	11	2	1,254
*p*(equal)	0.0007	<0.0001	0.0130	0.0044	0.0005			

*Within-subgroup estimates of effect sizes, and p-values from F-test for equality of effects across subgroups. n(e), number of effect sizes; k, number of studies; N, number of participants*.

Region and country of the study may also matter. For NR in particular, there is statistical evidence for variation across regions: Obs, NJ, and NR exhibited the largest effects in Europe, Des and Act in Asia. For all facets, the lowest effect estimates came from the three Latin American studies included (whereas studies done in Spain follow—and partly seem to cause—the European pattern of large effects for Obs, NJ, and NR). Nevertheless, these results should be interpreted with caution given the small number of studies outside North America. Similarly, the two studies done in the German language were suggestive that the language of the questionnaire might matter as they exhibited by far the largest effects for all facets (except Obs, where they show a very low effect). Details are available from the author.

Interestingly, for all facets except Obs and possibly NR, reported effects are smaller in papers that have at least some version easily available on the internet, compared to those that are not (Table 8).

**Table 8:** Desirable outcomes by publication availability.

	**Obs**	**Des**	**Act**	**NJ**	**NR**	***n*(e)**	***k***	***N***
Accessible	0.17	0.26	0.23	0.21	0.24	93	21	5,904
Paywall	0.15	0.37	0.36	0.36	0.30	51	22	5,667
*p*(equal)	0.6168	<0.0001	<0.0001	<0.0001	0.0650			

*Within-subgroup estimates of effect sizes, and p-values from F-test for equality of effects across subgroups. n(e), number of effect sizes; k, number of studies; N, number of participants*.

### Undesirable Outcomes

Results for the combined groups of clinical and unhelpful outcomes (144 effect sizes per facet) are as follows (Table 9):

**Table 9:** Meta-analysis for undesirable outcomes.

**Facet**	**All studies**								**Partial corr. available**			
	***r***	**Lower**	**Upper**	***p***	***Q***	**Q.pval**	**I_sq**	**tau_sq**	***r***	***p***	***r*(pc)**	***p*(pc)**
Obs	−0.02	−0.03	−0.01	0.0078	1,331.84	<0.0001	0.75	0.01	0.00	0.8852	0.01	0.4713
Des	0.19	0.18	0.21	<0.0001	1,567.82	<0.0001	0.79	0.01	0.20	<0.0001	0.10	<0.0001
Act	0.31	0.30	0.33	<0.0001	2,610.71	<0.0001	0.87	0.03	0.32	<0.0001	0.13	<0.0001
NJ	0.32	0.30	0.34	<0.0001	3,291.37	<0.0001	0.90	0.04	0.34	<0.0001	0.16	<0.0001
NR	0.20	0.19	0.22	<0.0001	1,924.08	<0.0001	0.83	0.02	0.23	<0.0001	0.14	<0.0001

*r, estimated correlation from random effects model; lower and upper, boundaries of the 95% confidence interval; p, p-value of r = 0; I_sq, I^2^ measure of heterogeneity; tau_sq, τ^2^ measure of heterogeneity; Q, measure of heterogeneity; Q.pval, p-value of Q = 0; r(pc), partial correlation; p(pc), p-value of r(pc) = 0*.

In contrast to the situation for desirable outcomes, the meta-analysis now showed that Obs had, if anything, a small negative effect, Des and NR had small and similar effects, Act and NJ had similar and medium sized effects. [Nevertheless, meta-analysis of the differences showed that the effects are also significantly different between NJ and Act (Δ*r* = 0.02, *p* = 0.0078), and between Des and NR (Δ*r* = 0.03, *p* < 0.0001)]. The latter four facets also exhibited small partial correlations ranging between *r* = 0.10 for Des and 0.16 for NJ, with the confidence intervals for Des and NJ disjoint.

Heterogeneity is a substantial concern, given that all *I*^2^ are greater than 0.75.

The results again seem quite robust with respect to publication bias. Even assuming extreme bias, the estimated effect sizes for Acting and Non-judging remain above 0.2 (Table 10).

**Table 10:** Robustness to publication bias for undesirable outcomes.

	**1-sided**					**2-sided**				
**Prob**	**Obs**	**Des**	**Act**	**NJ**	**NR**	**Obs**	**Des**	**Act**	**NJ**	**NR**
1	−0.02	0.19	0.30	0.31	0.20	−0.02	0.19	0.30	0.31	0.20
0.8	−0.03	0.19	0.30	0.31	0.20	−0.02	0.18	0.30	0.30	0.19
0.6	−0.04	0.19	0.30	0.30	0.20	−0.02	0.17	0.29	0.29	0.18
0.4	−0.06	0.19	0.30	0.30	0.19	−0.02	0.15	0.27	0.27	0.16
0.2	−0.10	0.18	0.30	0.30	0.19	−0.01	0.13	0.25	0.25	0.14
0.1	−0.14	0.18	0.30	0.30	0.19	−0.01	0.12	0.24	0.24	0.13
0.05	−0.18	0.18	0.30	0.30	0.19	−0.01	0.11	0.23	0.23	0.12

*Prob, probability that a result with p-value less than 0.05 gets published*.

In contrast to the case of desirable outcomes, meta-regressions resulted in coefficients that were too small to be practically meaningful even in the few cases where the associated *p*-values were below 0.05. Grouping into clinical, non-clinical, and mixed samples resulted in considerable evidence against equality of effects (*p* < 0.0001 for three facets), but this seems to be driven mostly by the one mixed study, with the estimates for the purely clinical and purely non-clinical samples quite similar. In a like manner, and partly in line with the results for desirable outcomes, the strongest effects were found for the three mixed (meditating and non-meditating) samples for Des, Act, and NJ, whereas the strongest effects for Obs and NR were among meditators (though the *p*-value for overall difference in subgroups effects was statistically insignificant in the latter case).

Dependence on occupation for undesirable outcomes was attenuated compared to the case of desirable outcomes (though still statistically significant: *p* = 0.0220 for Act, *p* = 0.0271 for NJ and *p* < 0.0001 for the other facets). In particular, the extreme results for Academic and Professional samples were not replicated here. Neither did the extreme results above concerning the region in which research was conducted hold here. Only for NR was there any evidence for differences between regions (*p* = 0.0042), again with Europe showing the strongest and Latin America the weakest effects. For both grouping according to occupation, and according to region, each group contained at least five studies, thus sample size should be less of a concern than for the corresponding results for desirable outcomes. Concerning questionnaire language, there was again some evidence that it matters for the outcome, in this case for NJ (*p* = 0.0010). Nevertheless, direct comparison to the results for desirable outcomes is not possible since that sample contained German but no French studies, whereas here the case is reversed.

Interestingly, the free availability of a publication now appeared to bear little relationship with the estimated effects, with only Act showing some evidence of being lower in openly accessible studies (*r* = 0.30 vs. 0.35, *p* = 0.0302). Finally, the choice between subjective and objective outcome measures matters: Estimates were much smaller for objective measures for Act, NJ, NR (all *p* < 0.0001). Surprisingly, the estimate for Obs for objective measures was larger (*r* = 0.16) than for subjective ones (*r* = −0.02) with a *p*-value of 0.0156.

### Further Categories

The number of effect sizes in the categories of physiological outcomes (labeled “body”) and social outcomes were much smaller, thus I report only the main results briefly: The effects in the “body” category turned out to be very small and in three of the five cases statistically insignificant, the larger ones were for Act (*r* = 0.1110, *p* = 0.0354) and NJ (*r* = 0.0988, *p* = 0.0060). Heterogeneity was here less severe than with other groupings. Remarkably, some partial correlations were higher than zero-order correlations, this being most pronounced for NR (Act: *r* = 0.18, *p* < 0.0001, NJ: *r* = 0.21, *p* = 0.0076, NR: *r* = 0.17, *p* = 0.0010). Effect estimates for social outcomes were small (ranging from *r* = 0.1 for Obs to *r* = 0.21 for NJ, all *p* < 0.0003), with the largest partial correlation shown by NR (*r* = 0.16, *p* < 0.0001).

## Discussion

This paper contributes to the “exploration [of links] between specific DM facets and psychological health” (Tomlinson et al., 2018), and between those facets and well-being, by meta-analytically studying the strength, statistical significance, and robustness of correlations; by evaluating possible moderators; and by estimating the effects of possible publication bias.

It differs from the existing literature in a number of important ways: (a) Existing systematic reviews and meta-analyses of the effects of mindfulness tend to lump various measures of mindfulness together (sometimes even neglecting to list the specific mindfulness measures used in the studies they survey, e.g., Lomas et al., 2017); in contrast, the present work is restricted to one particular, and widely-used, measure of mindfulness (the FFMQ). (b) Despite its obvious importance for clarifying the concept of mindfulness, virtually no meta-analysis compared different facets of mindfulness. One exception is Quaglia et al. (2016), but there again similar-looking facets from different measures were lumped together. In this respect too, the present paper avoids confounding different measures. (c) A pre-specified primary outcome measure was used, thereby reducing ambiguity in reporting results. (d) Publication bias was dealt with using a technique that is appropriate given the high level of heterogeneity in most outcomes (this heterogeneity was also found in the existing literature, e.g., Quaglia et al., 2016—who nevertheless used the—in this case problematic—techniques of forest plots and trim-and-fill to deal with the question of publication bias).

### Strength and Robustness of Correlations

Across conditions, Describe, Act-with-awareness, Non-judge and (to a lesser extent) Non-react were moderately and non-redundantly correlated with outcomes: Under the widely used rules of thumb to classify correlations according to strength into small (*r* between 0.1 and 0.3), medium (*r* between 0.3 and 0.5), and large (*r* > 0.5) correlations, only Non-judging exhibited large correlations (with AAQ-II and PWB). Medium sized correlations above 0.4 were revealed for Describing with “Other eudaimonia” [due to strong correlations with certain subscales of PWB reported in two papers: Bravo et al. (2016) and Bergin and Pakenham (2016)] and with PWB itself, for Acting-with-awareness with PWB, AAQ-2, and the (reversed) scales BDI-2, PANAS.NA, and PSS, for Non-judging (with reversed DASS, PANAS.NA, PSS, PSWQ) and for Non-reacting with PWB and AAQ-2. In addition, there was a considerable number of correlations between 0.3 and 0.4 for all facets except Observing.

These effects seemed quite robust to taking subsamples, and to reasonable rates of publication bias (if, say, at least 20% of statistically insignificant results get published). Nevertheless, one should always keep in mind that the guidelines for classifying correlation strength are only very rough rules of thumb and the actual importance of a given effect size can vary widely depending on circumstances. Also, precision of estimates is negatively impacted by heterogeneity and a lack of repeated use of measures.

### Discussion by Facet

The Observe facet showed the expected dependence on meditation experience that was found in previous publications. Nevertheless, the Observe facet turned out to have small, but non-trivial, correlations with measures of desirable outcomes even for non-meditators. Therefore, it might be premature to simply drop this scale from the FFMQ when applied to non-meditators, as some authors advocate (e.g., Duan, 2016), and it should be interesting to study correlations of positive outcomes with the factors found in Rudkin et al. (2018).

The Describe facet had the highest zero-order correlation with the category of all desirable outcomes, which nevertheless did not hold for partial correlations. In addition, it had correlations >0.25 with the other categories of desirable outcomes studied, and correlations mostly around 0.2 with (reversed) undesirable outcomes. In particular, it showed the relatively largest (but still small, and imprecisely measured) correlation with outcomes classified as “social” (measures like the Compassionate Love Scale, or the Agreeableness and Extroversion sub-scales of the Big Five), which may not be too surprising since a high score on Describe could indicate a general ability to communicate, which in turn may be helpful in social contexts independently of mindfulness (for example, one item on this subscale of the FFMQ reads “I can easily put my beliefs, opinions, and expectations into words”).

Acting-with-awareness generally tended to be among the facets showing the highest correlation with outcomes. Its estimated correlation with the primary measure in this meta-analysis, SWLS, and with the reversed depression scale, seemed to be the strongest among the five facets, but these estimates were too imprecise (because of small samples, heterogeneity, and at least for SWLS because of an apparent outlier in the data) to allow firm conclusions. In any case, a correlation of Act with absence of depression would not be surprising since the items on the Act scale pertain to the ability to concentrate and actually do something; hence, depression might conceivably lead to a low score on Act (rather than this mindfulness facet relieving depression).

As noted above, Non-judging distinguished itself as being the only facet exhibiting strong correlations with some outcomes. It also had the strongest correlation with the absence of undesirable outcomes. Generally, it tended to be among the most correlated facets.

The Non-reacting facet tended to have somewhat lower correlations with outcomes than Act and NJ, and in several cases than Des. Interestingly though, it may have more of a unique contribution to outcomes than zero-order correlations indicate, as it has in several cases relatively higher partial correlations. In particular, it exhibits the only close to medium sized partial correlation [*r* = 0.29 with reversed PSWQ, 95% confidence interval (0.13, 0.43)].

### Publication Bias

One of the main strengths of the present work is that it deals with publication bias in a way that is suitable given the substantial heterogeneity revealed in the meta-analyses. As noted above, calculations by Coronado-Montoya et al. (2016) implied that only about 27.3% of insignificant results get published. This estimate was based on an assumed true effect size of *d* = 0.55 which corresponds to a correlation of *r* = 0.265, an effect size which is well-compatible with the meta-analytic effect size estimates presented here. Therefore, it is reassuring that the results of these meta-analyses proved to be reasonably robust under even more severe publication bias in which only 10% of statistically insignificant results get published. Furthermore, this still holds under alternative assumptions for the true effect size; for example, assuming a very small effect of *r* = 0.1 (corresponding to *d* = 0.2) leads to an estimated publication rate of 14.8% for insignificant results using the Coronado-Montoya methodology, which is still compatible with robustness.

### Moderators

This study found a number of additional moderators, in addition to the well-known meditators vs. non-meditators distinction for the Observe facet. Estimates suggested that these are not only of high statistical significance but that their influence is of practically relevant magnitude, as will be discussed below separately for regressions and subgroups.

#### Regressions

The effect of publication year as well as the composition of the sample (age, sex) turned out to be of non-negligible size: Given that the correlation of NR with Other_Eudaimonia was estimated above at *r* = 0.22, the estimated regression coefficient implied that a change of 23.45 percentage points in the proportion of men in the sample seems to reduce this effect to 0. Similarly, an increase of the mean age in the sample of 38 years would turn the estimated effect of *r* = −0.04 in a medium sized positive correlation above *r* = 0.3.

#### Subgroups

There are several statistically significant (even after Bonferroni-adjusting for 400-fold testing: 8 planned groups ^*^ 5 facets ^*^ 10 moderators) and large effects with change the differences in correlation estimates between subgroups up to almost 0.4 (which means a medium sized effect would be turned into zero!).

Perhaps most interestingly, there is considerable evidence that not only the Observe facet behaves differently among meditators, but also all the other facets (for at least some outcomes). Nevertheless, these differences are difficult to interpret since the most extreme values are in the “mixed” groups. As discussed in the above, there is also considerable evidence for a difference in results between objective and subjective outcome measures, in particular for clinical outcomes. In addition, the occupation of participants seemed to influence the outcomes for negative results for Obs and Des, with the largest by far effects estimates obtained in the university samples. The country in which research is conducted may matter too, but there seems no clear pattern in the results appearing. Finally, there may be differences according to publication quality, but these estimates were considerably smaller than those mentioned before.

#### Caveat

Interesting as these results are, many of the significant relationships arose in situations where at least some subgroups contained results from few (often only two) samples and in addition a small number of effect sizes. This and the occasionally erratic-looking underlying patterns suggest caution in interpreting these results.

## Conclusions

With respect to one of the two main aims of the present paper, a straightforward conclusion can be drawn from the results reported above: Publication bias is unlikely to seriously distort the beneficial effects of mindfulness as presented in the literature.

The second main aim was to study facets of mindfulness and to relate them both to conceptualizations of mindfulness and to intervention research. Concerning intervention design, the importance of the Non-judgement facet showed itself clearly in this meta-analysis, but even the Observe facet proved itself valuable for achieving positive (in contrast to avoiding negative) outcomes and therefore should not be neglected in interventions. Concerning research into the effectiveness of interventions, the present results point to the necessity of distinguishing participants not only according to their meditation experience, but also by their professional background and possibly by home country.

Conceptualizations of mindfulness cannot be adequately discussed in the space available here. Nevertheless, I do want to suggest two possible conclusions: On the one hand, to the extent that one is primarily interested in a more immediate problem-solution-focused approach which is maybe more in line with most “secular mindfulness” applications studied in contemporary psychological research, the Non-judgement facet stands out in regard to clinical applications, whereas the contributions of the facets may be more balanced in regard to the positive-psychology-type outcome measures that have been used up to now. Concerning the Buddhist roots of mindfulness, it seems worth recalling that the Buddha's quest was motivated by existential concerns: birth, the unavoidability of disease, aging, and death once you are born, and the impermanence of positive states—aspects of the human experience that are unalterable and therefore have to be accepted. This again points to the importance of non-judging; it may also help build a bridge between existential and more immediately therapeutic concerns (compare, e.g., Noyon and Heidenreich, 2012; Iverach et al., 2014; Tichy, 2018).

### Limitations

This study has a number of limitations. First of all, the present work looked only at correlations and therefore does not allow conclusions concerning causality. Second, assigning outcomes to the groups is both subjective and error prone (even though the fact that results generally held up in subsamples should mitigate concerns in this direction somewhat); for example, Goodman et al. (2017) provided a strong argument against distinguishing between hedonia and eudaimonia, so that the distinction made above may actually be vacuous. Third, some of the resulting groups are small, especially when trying to test possible moderators via subgroup comparisons. In particular, the number of not purely subjective effects included is small. Fourth, the issue of multiple testing is a complicated one in that it is often not clear what the correct number of tests to adjust for is (e.g., should regressions and subgroup comparisons for moderators be treated separately—as done here—or combined?).

### Further Research

A wide range of future research suggests itself as desirable, as in part already discussed above. Particularly interesting seems studying the relationship between mindfulness and its facets on one hand, and judgement and decision making on the other. As argued in the introduction, some might consider it counter-intuitive that non-judgement and non-reactivity would contribute to positive outcomes; but the present results show that the Non-judgement facet is the only one that has strong correlations with any of these outcomes, and Non-reactivity seems to provide an interesting unique contribution.

Noticeably, the facets most strongly correlated with outcomes tend to be the ones whose items are all (Act, NJ) or partly (Des) negatively worded, whereas the less correlated NR and Obs are entirely positively worded. This raises the question of whether these differences in estimated effect sizes are due to the wording. This seems plausible since, on the one hand, for the FFMQ, Dam et al. (2009) showed that “meditators and non-meditators with similar overall levels of mindfulness differentially endorse response options for positively and negatively worded items”; and on the other hand, negative and positive items are known to function differently in the Self-Compassion Scale (SCS): Muris and Petrocchi (2016) found “stronger effects for the negative SCS sub-scales (*r* range from 0.47 to 0.50) than for the positive SCS sub-scales (*r* range from −0.27 to −0.34),” similar results for the German version of the SCS were obtained in Coroiu et al. (2018).

In any case, the present results do provide strong evidence that Non-judging and Non-reacting are correlated with positive outcomes. Consistent with this, the conceptually related construct of Non-attachment is more strongly correlated with well-being than any of the FFMQ facets (Sahdra et al., 2016), and un-clinging may be more beneficial than awareness (Ng et al., 2017). Also consistent with this are the results of Lebuda et al. (2015), whose meta-analysis of 89 correlations found a stronger connection of creativity with the open monitoring aspect than with the awareness aspect of mindfulness. It is also worth pointing out that, even though Buddhist orthodoxy seems to distinguish between right and wrong views, it has also been argued that the Buddha may have considered all views as problematic (e.g., Fuller, 2005).

Clearly, the connection of these concepts with the psychology of judgement and decision making deserves closer scrutiny: Currently, a number of studies explored the relationship between mindfulness (in most cases measured using the unidimensional Mindful Attention Awareness Scale—MAAS) with executive control, attention, and memory (for an overview see for example, Sun et al., 2015). Few studies have looked at the relation to cognitive illusions (Pohl, 2005; Kahneman, 2011), and those that exist do not seem to arrive at a clear picture. For example, related to the sunk cost bias, Hafenbrack et al. (2014) argued that the present focus and reduced negativity in mindfulness led to attenuation of the sunk-cost bias, whereas Schmitzer-Torbert (2018) argue that “the relationship between trait mindfulness and sunk-costs is weaker [than that with escalation of commitment] and inconsistent.” Concerning motivated perception, Nickerson and Brown (2016) criticize the size of effects claimed in Adair and Fredrickson (2015). Concerning ethical decision making, Shapiro et al. (2012) argued that participation in an MBSR course “resulted in improvements in moral reasoning and ethical decision making,” whereas Mattes (2018) effectively argued that such effects are small and may be compensated by increased overconfidence into judgements, so that mindfulness can easily coexist with ethical dogmatism.

Future work should include repeated use of outcome measures like SWLS, to strengthen the results and reduce the impact of outliers, and to further investigate the factor structure of the FFMQ in various populations (Tran et al., 2013, is one example of such a study). On the other hand, similar analyses with other multi-dimensional measures of mindfulness (in particular measures with less imbalance between positively and negatively worded items) seem crucial both for clarifying the role of mindfulness in positive and clinical psychology, and for clarifying the concept(s) of mindfulness itself.

## Data Availability Statement

Data and scripts used can be downloaded at the authors homepage and are included as Supplementary Material.

## Author Contributions

The author confirms being the sole contributor of this work and has approved it for publication.

### Conflict of Interest

The author declares that the research was conducted in the absence of any commercial or financial relationships that could be construed as a potential conflict of interest.
