# Supplementary material for: Systematic Review and Meta-Analysis of Correlates of FFMQ Mindfulness Facets
Source: Front Psychol. 2019 Dec 6;10:2684. doi: 10.3389/fpsyg.2019.02684 (PMC6909938; doi:10.3389/fpsyg.2019.02684)
Supplement: Supplementary file 1 [file Table_1.docx]

### Table 1: List of papers included in the meta-analysis

Adam, F., Heeren, A., Day, J. and de Sutter, P. (2015). Development of the sexual Five-Facet Mindfulness Questionnaire (FFMQ-S): Validation among a community sample of French-speaking women.. Journal of Sex Research, 52, 617-626.

Alda, M., Puebla-Guedea, M., Rodero, B., Demarzo, M., Montero-Marin, J., Roca, M. and Garcia-Campayo, J. (2016). Zen meditation, length of telomeres, and the role of experiential avoidance and compassion. Mindfulness, 7(3), 651-659.

Antonova, E., Amaratunga, K., Wright, B. and Kumari, V. (2016). Schizotypy and mindfulness: Magical thinking without suspiciousness characterizes mindfulness meditators. Schizophrenia Research: Cognition, 5, 1-6.

Baer, R. A., Lykins, E. L. B. and Peters, J. R. (2012). Mindfulness and self-compassion as predictors of psychological wellbeing in long-term meditators and matched nonmeditators.. The Journal of Positive Psychology, 7(3), 230-238.

Baer, R. A., Smith, G. T., Lykins, E., Button, D., Krietemeyer, J., Sauer, S., Walsh, E., Duggan, D. and Williams, J. M. G. (2008). Construct validity of the Five Facet Mindfulness Questionnaire in meditating and nonmeditating samples.. Assessment, 15(3), 329-342.

Barnes, S. M. and Lynn, S. J. (2010). Mindfulness skills and depressive symptoms: A longitudinal study.. Imagination, Cognition and Personality, 77-91.

Bergin, A. J. and Pakenham, K. I. (2016). The stress-buffering role of mindfulness in the relationship between perceived stress and psychological adjustment.. Mindfulness, 7(4), 928-939.

Biglan, A., Layton, G. L., Jones, L. B., Hankins, M. and Rusby, J. C. (2013). The value of workshops on psychological flexibility for early childhood special education staff.. Topics in Early Childhood Special Education, 32(4), 196-210.

Boden, M. T., Irons, J. G., Feldner, M. T., Bujarski, S. and Bonn-Miller, M. O. (2015). An investigation of relations among quality of life and individual facets of emotional awareness and mindfulness.. Mindfulness, 6(4), 700-707.

Bodenlos, J. S., Wells, S. Y., Noonan, M. and Mayrsohn, A. (2015). Facets of dispositional mindfulness and health among college students.. The Journal of Alternative and Complementary Medicine, 21(10), 645-652.

Bowlin, S. L. and Baer, R. A. (2012). Relationships between mindfulness, self-control, and psychological functioning.. Personality and Individual Differences, 52(3), 411-415.

Bravo, A. J., Boothe, L. G. and Pearson, M. R. (2016). Getting personal with mindfulness: A latent profile analysis of mindfulness and psychological outcomes.. Mindfulness, 7(2), 420-432.

Brown, D. B., Bravo, A. J., Roos, C. R. and Pearson, M. R. (2015). Five facets of mindfulness and psychological health: Evaluating a psychological model of the mechanisms of mindfulness.. Mindfulness, 6(5), 1021-1032.

Caluyong, M. B., Zambrana, A. F., Romanow, H. C., Nathan, H. J., Nahas, R. and Poulin, P. A. (2015). The relationship between mindfulness, depression, diabetes self-care, and health-related quality of life in patients with type 2 diabetes.. Mindfulness, 6(6), 1313-1321.

Camilleri, G. M., Méjean, C., Bellisle, F., Hercberg, S. and Péneau, S. (2015). Association between Mindfulness and Weight Status in a General Population from the NutriNet-Santé Study. PLOS ONE, 10, e0127447.

Campos, D., Botella, C., Quero, S. and Cebolla, A. (2015). Meditation and happiness: Mindfulness and self-compassion may mediate the meditation-happiness relationship. Personality and Individual Differences, 93, 80-85.

Cebolla, A., Garcia-Palacios, A., Soler, J., Guillen, V., Banos, R. and Botella, C. (2012). Psychometric properties of the Spanish validation of the Five Facets of Mindfulness Questionnaire (FFMQ). European Journal of Psychiatry, 26, 118-126.

Christopher, M. S., Neuser, N. J., Michael, P. G. and Baitmangalkar, A. (2012). Exploring the psychometric properties of the Five Facet Mindfulness Questionnaire.. Mindfulness, 3(2), 124-131.

Consedine, N. S. and Butler, H. F. (2014). Mindfulness, health symptoms and healthcare utilization: Active facets and possible affective mediators.. Psychology, Health & Medicine, 19(4), 392-401.

Corthorn, C. and Milicic, N. (2016). Mindfulness and parenting: A correlational study of non-meditating mothers of preschool children.. Journal of Child and Family Studies, 25(5), 1672-1683.

Curtiss, J. and Klemanski, D. H. (2014a). Factor analysis of the Five Facet Mindfulness Questionnaire in a heterogeneous clinical sample.. Journal of Psychopathology and Behavioral Assessment, 36(4), 683-694.

Curtiss, J. and Klemanski, D. H. (2014b). Teasing apart low mindfulness: Differentiating deficits in mindfulness and in psychological flexibility in predicting symptoms of generalized anxiety disorder and depression.. Journal of Affective Disorders, 166, 41-47.

Day, M. A., Smitherman, A., Ward, L. C. and Thorn, B. E. (2015). An investigation of the associations between measures of mindfulness and pain catastrophizing.. The Clinical Journal of Pain, 31(3), 222-228.

Desrosiers, A., Klemanski, D. H. and Nolen-Hoeksema, S. (2013). Mapping mindfulness facets onto dimensions of anxiety and depression. Behavior Therapy, 44(3), 373-384.

Elices, M., Pascual, J. C., Carmona, C., Martín-Blanco, A., Feliu-Soler, A., Ruiz, E., Gomà-i-Freixanet, M., Pérez, V. and Soler, J. (2015). Exploring the relation between childhood trauma, temperamental traits and mindfulness in borderline personality disorder. BMC psychiatry, 15, 180.

Emanuel, A. S., Updegraff, J. A., Kalmbach, D. A. and Ciesla, J. A. (2010). The role of mindfulness facets in affective forecasting.. Personality and Individual Differences, 49(7), 815-818.

Evans, D. R., Baer, R. A. and Segerstrom, S. C. (2009). The effects of mindfulness and self-consciousness on persistence.. Personality and Individual Differences, 47(4), 379-382.

Evans, D. R. and Segerstrom, S. C. (2011). Why do mindful people worry less?. Cognitive Therapy and Research, 35(6), 505-510.

Fatter, D. M. and Hayes, J. A. (2013). What facilitates countertransference management? The roles of therapist meditation, mindfulness, and self-differentiation.. Psychotherapy Research, 23(5), 502-513.

Fernandez, A. C., Wood, M. D., Stein, L. A. R. and Rossi, J. S. (2010). Measuring mindfulness and examining its relationship with alcohol use and negative consequences.. Psychology of Addictive Behaviors, 24(4), 608-616.

Fink, S., Foran, K. A., Sweeney, A. C. and O'Hea, E. L. (2009). Sexual body esteem and mindfulness in college women.. Body Image, 6(4), 326-329.

Frank, D. J., Nara, B., Zavagnin, M., Touron, D. R. and Kane, M. J. (2015). Validating older adults' reports of less mind-wandering: An examination of eye movements and dispositional influences.. Psychology and Aging, 30(2), 266-278.

Fuller, N. R., Sainsbury, A., Caterson, I. D., Enarsson, M., Denyer, G., Manns, C., Swinbourne, J., da Luz, F. Q., Fong, M. and Markovic, T. P. (2016). Examining mindfulness as a predictor of weight loss--Findings from the DIABEGG study. Obesity Research & Clinical Practice, 11, 88-96.

Garland, E. L., Boettiger, C. A., Gaylord, S., Chanon, V. W. and Howard, M. O. (2012). Mindfulness is inversely associated with alcohol attentional bias among recovering alcohol-dependent adults. Cognitive Therapy and Research, 36(5), 441-450.

Garland, E. L. and Roberts-Lewis, A. (2013). Differential roles of thought suppression and dispositional mindfulness in posttraumatic stress symptoms and craving. Addictive Behaviors, 38(2), 1555-1562.

Garland, S. N., Campbell, T., Samuels, C. and Carlson, L. E. (2013). Dispositional mindfulness, insomnia, sleep quality and dysfunctional sleep beliefs in post-treatment cancer patients.. Personality and Individual Differences, 55(3), 306-311.

Gonzalez, A., Locicero, B., Mahaffey, B., Fleming, C., Harris, J. and Vujanovic, A. A. (2016). Internalized HIV stigma and mindfulness: Associations with PTSD symptom severity in trauma-exposed adults with HIV/AIDS.. Behavior Modification, 40(1-2), 144-163.

Goodall, K., Trejnowska, A. and Darling, S. (2012). The relationship between dispositional mindfulness, attachment security and emotion regulation. Personality and Individual Differences, 52(5), 622-626.

Gorbovskaya, I., Park, N. and Kim, C. (2014). The Relationships Between Mindfulness, Distraction Control, and Working Memory. Inkblot: The Undergraduate Journal of Psychology, .

Gu, J., Strauss, C., Crane, C., Barnhofer, T., Karl, A., Cavanagh, K. and Kuyken, W. (2016). Examining the factor structure of the 39-item and 15-item versions of the Five Facet Mindfulness Questionnaire before and after mindfulness-based cognitive therapy for people with recurrent depression.. Psychological assessment, 28, 791-802.

Hamill, T. S., Pickett, S. M., Amsbaugh, H. M. and Aho, K. M. (2015). Mindfulness and acceptance in relation to Behavioral Inhibition System sensitivity and psychological distress.. Personality and Individual Differences, 72, 24-29.

Hanley, A. W. and Garland, E. L. (2014). Dispositional mindfulness co-varies with self-reported positive reappraisal.. Personality and Individual Differences, 66, 146-152.

Hanley, A. W., Palejwala, M. H., Hanley, R. T., Canto, A. I. and Garland, E. L. (2015). A failure in mind: Dispositional mindfulness and positive reappraisal as predictors of academic self-efficacy following failure.. Personality and Individual Differences, 86, 332-337.

Heeren, A., Douilliez, C., Peschard, V. and Philippot, P. (2011). Cross-cultural consistency of the Five Facets Mindfulness Questionnaire: adaptation and validation in a French sample. European Review of Applied Psychology, 61, 147-151.

Hollis-Walker, L. and Colosimo, K. (2011). Mindfulness, self-compassion, and happiness in non-meditators: A theoretical and empirical examination.. Personality and Individual Differences, 50(2), 222-227.

Huston, D. C., Garland, E. L. and Farb, N. A. S. (2011). Mechanisms of mindfulness in communication training.. Journal of Applied Communication Research, 39(4), 406-421.

Jennings, P. A. (2015). Early childhood teachers' well-being, mindfulness, and self-compassion in relation to classroom quality and attitudes towards challenging students.. Mindfulness, 6(4), 732-743.

Johns, K. N., Allen, E. S. and Gordon, K. C. (2015). The relationship between mindfulness and forgiveness of infidelity.. Mindfulness, 6(6), 1462-1471.

Jones, K. D., Mist, S. D., Casselberry, M. A., Ali, A. and Christopher, M. S. (2015). Fibromyalgia Impact and Mindfulness Characteristics in 4986 People with Fibromyalgia.. Explore, 11, 304-309.

Jones, S. M. and Hansen, W. (2015). The impact of mindfulness on supportive communication skills: Three exploratory studies.. Mindfulness, 6(5), 1115-1128.

Kalill, K. S., Treanor, M. and Roemer, L. (2014). The importance of non-reactivity to posttraumatic stress symptoms: A case for mindfulness.. Mindfulness, 5(3), 314-321.

Karyadi, K. A. and Cyders, M. A. (2015). Elucidating the association between trait mindfulness and alcohol use behaviors among college students.. Mindfulness, 6(6), 1242-1249.

Keane, A. (2014). The influence of therapist mindfulness practice on psychotherapeutic work: A mixed-methods study.. Mindfulness, 5(6), 689-703.

Kearney, D. J., Malte, C. A., McManus, C., Martinez, M. E., Felleman, B. and Simpson, T. L. (2013). Loving-kindness meditation for posttraumatic stress disorder: A pilot study.. Journal of Traumatic Stress, 26(4), 426-434.

Khaddouma, A., Gordon, K. C. and Bolden, J. (2015). Zen and the art of sex: Examining associations among mindfulness, sexual satisfaction, and relationship satisfaction in dating relationships.. Sexual and Relationship Therapy, 30(2), 268-285.

Kraemer, K. M., McLeish, A. C. and Johnson, A. L. (2015). Associations between mindfulness and panic symptoms among young adults with asthma. Psychology, Health & Medicine, 20, 322-331.

Lara, R., Herrero, M., Blanco-Donoso, L. and Chavez, A. (2015). Propiedades psicometricas del "cuestionario de cinco facetas de la conciencia plena" (five facet mindfulness questionnaire, FFMQ-M) en Mexico. Psicología Conductual, 23(3), 467-487.

Lattimore, P., Fisher, N. and Malinowski, P. (2011). A cross-sectional investigation of trait disinhibition and its association with mindfulness and impulsivity. Appetite, 56(2), 241-248.

Lavender, J. M., Gratz, K. L. and Tull, M. T. (2011). Exploring the Relationship between Facets of Mindfulness and Eating Pathology in Women.. Cognitive Behaviour Therapy, 40(3), 174-182.

Lee, A. C., Harvey, W. F., Price, L. L., Morgan, L. P. K., Morgan, N. L. and Wang, C. (2016). Mindfulness is associated with psychological health and moderates pain in knee osteoarthritis.. Osteoarthritis and cartilage / OARS, Osteoarthritis Research Society, .

Levin, M. E., Dalrymple, K., Himes, S. and Zimmerman, M. (2014a). Which facets of mindfulness are related to problematic eating among patients seeking bariatric surgery?. Eating Behaviors, 15(2), 298-305.

Levin, M. E., Dalrymple, K. and Zimmerman, M. (2014b). Which facets of mindfulness predict the presence of substance use disorders in an outpatient psychiatric sample?. Psychology of Addictive Behaviors, 28(2), 498-506.

Lustyk, M. K. B., Gerrish, W. G., Douglas, H., Bowen, S. and Marlatt, G. A. (2011). Relationships among premenstrual symptom reports, menstrual attitudes, and mindfulness.. Mindfulness, 2(1), 37-48.

Michalak, J., Zarbock, G., Drews, M. and Heidenreich, T. (2016). Erfassung von Achtsamkeit mit der deutschen Version des Five Facet Mindfulness Questionnaires (FFMQ-D). Zeitschrift für Gesundheitspsychologie, 24, 1-12.

Mira, A., Campos, D., Etchemendy, E. and Cebolla, A. (2016). Access to autobiographical memory as an emotion regulation strategy and its relation to dispositional mindfulness. Mindfulness & Compassion, 1, 39-44.

Montgomery, K., Norman, P., Messenger, A. G. and Thompson, A. R. (2016). The importance of mindfulness in psychosocial distress and quality of life in dermatology patients. British Journal of Dermatology, 175, 930-936.

Murphy, C. and MacKillop, J. (2012). Living in the here and now: Interrelationships between impulsivity, mindfulness, and alcohol misuse. Psychopharmacology, 219(2), 527-536.

Neale-Lorello, D. and Haaga, D. A. F. (2015). The "observing" facet of mindfulness moderates stress/symptom relations only among meditators. Mindfulness, 6(6), 1286-1291.

Ostafin, B. D., Brooks, J. J. and Laitem, M. (2014). Affective reactivity mediates an inverse relation between mindfulness and anxiety.. Mindfulness, 5(5), 520-528.

Pearson, M. R., Lawless, A. K., Brown, D. B. and Bravo, A. J. (2015). Mindfulness and emotional outcomes: Identifying subgroups of college students using latent profile analysis. Personality and Individual Differences, 76, 33-38.

Pepping, C. A., O'Donovan, A. and Davis, P. J. (2013). The positive effects of mindfulness on self-esteem. The Journal of Positive Psychology, 8(5), 376-386.

Pepping, C. A., O'Donovan, A. and Davis, P. J. (2014). The differential relationship between mindfulness and attachment in experienced and inexperienced meditators. Mindfulness, 5(4), 392-399.

Peters, J. R., Eisenlohr-Moul, T. A., Upton, B. T. and Baer, R. A. (2013). Nonjudgment as a moderator of the relationship between present-centered awareness and borderline features: Synergistic interactions in mindfulness assessment.. Personality and Individual Differences, 55(1), 24-28.

Peters, J. R., Erisman, S. M., Upton, B. T., Baer, R. A. and Roemer, L. (2011). A preliminary investigation of the relationships between dispositional mindfulness and impulsivity. Mindfulness, 2(4), 228-235.

Peters, J. R., Smart, L. M., Eisenlohr-Moul, T. A., Geiger, P. J., Smith, G. T. and Baer, R. A. (2015). Anger rumination as a mediator of the relationship between mindfulness and aggression: The utility of a multidimensional mindfulness model. Journal of Clinical Psychology, 71(9), 871-884.

Quezada-Berumen, L. D., Gonzalez-Ramirez, M. T., Cebolla, A., Soler, J. and Garcia-Campayo, J. (2014). Conciencia corporal y mindfulness: Validacion de la version Espanola de la Escala de Conexion Corporal (SBC). / Body awareness and mindfulness: Validation of the Spanish version of the Scale of Body Connection. Actas Espanolas De Psiquiatria, 42(2), 57-67.

Raphiphatthana, B., Jose, P. E. and Kielpikowski, M. (2016). How do the facets of mindfulness predict the constructs of depression and anxiety as seen through the lens of the tripartite theory? Personality and Individual Differences, 93, 104-111.

Reese, E. D., Zielinski, M. J. and Veilleux, J. C. (2015). Facets of mindfulness mediate behavioral inhibition systems and emotion dysregulation. Personality and Individual Differences, 72, 41-46.

Rice, V., Boykin, G., Jeter, A. and Alfred, P. (2013). The Relationship between Mindfulness and Resiliency among Active Duty Service Members and Military Veterans. In: Proceedings of the Human Factors and Ergonomics Society Annual Meeting.

Roos, C. R., Pearson, M. R. and Brown, D. B. (2015). Drinking motives mediate the negative associations between mindfulness facets and alcohol outcomes among college students. Psychology of Addictive Behaviors, 29(1), 176-183.

Royuela-Colomer, E. and Calvete, E. (2016). Mindfulness facets and depression in adolescents: Rumination as a mediator. Mindfulness, 7(5), 1092–1102.

Ruiz, F. J., Odriozola-Gonzalez, P. and Suarez-Falcon, J. C. (2014). The Spanish version of the Believability of Anxious Feelings and Thoughts Questionnaire. Psicothema, 26(3), 308-313.

Schmidt, C. and Vinet, E. V. (2015). Atención Plena: Validación del Five Facet Mindfulness Questionnaire (FFMQ) en estudiantes universitarios chilenos / Mindfulness: Validation of the Five Facet Mindfulness Questionnaire (FFMQ) in Chilean university students. Terapia Psicologica, 33(2), 93-101.

Seli, P., Carriere, J. S. A. and Smilek, D. (2015). Not all mind wandering is created equal: dissociating deliberate from spontaneous mind wandering. Psychological research, 79, 750-758.

Shorey, R. C., Seavey, A. E., Quinn, E. and Cornelius, T. L. (2014). Partner-specific anger management as a mediator of the relation between mindfulness and female perpetrated dating violence. Psychology of Violence, 4(1), 51-64.

Short, M. M. and Mazmanian, D. (2013). Perfectionism and negative repetitive thoughts: Examining a multiple mediator model in relation to mindfulness. Personality and Individual Differences, 55(6), 716-721.

Short, M. M., Mazmanian, D., Oinonen, K. and Mushquash, C. J. (2016). Executive function and self-regulation mediate dispositional mindfulness and well-being. Personality and Individual Differences, 93, 97-103.

Slonim, J., Kienhuis, M., Di Benedetto, M. and Reece, J. (2015). The relationships among self-care, dispositional mindfulness, and psychological distress in medical students. Medical education online, 20.

Soler, J., Franquesa, A., Feliu-Soler, A., Cebolla, A., García-Campayo, J., Tejedor, R., Demarzo, M., Baños, R., Pascual, J. C. and Portella, M. J. (2014). Assessing decentering: Validation, psychometric properties, and clinical usefulness of the Experiences Questionnaire in a Spanish sample. Behavior Therapy, 45(6), 863-871.

Soler, J., Soriano, J., Ferraz, L., Grasa, E., Carmona, C., Portella, M. J., Seto, V., Alvarez, E. and Pérez, V. (2013). Direct experience and the course of eating disorders in patients on partial hospitalization: A pilot study. European Eating Disorders Review, 21(5), 399-404.

Spofford, J. L., Nevels, R. M., Gontkovsky, S. T. and Bell, T. P. (2014). Meditative practices predict spirituality but mindfulness does not predict alcohol use in African-American college students. Mental Health Religion & Culture, , 379-389.

Tabak, N. T., Horan, W. P. and Green, M. F. (2015). Mindfulness in schizophrenia: Associations with self-reported motivation, emotion regulation, dysfunctional attitudes, and negative symptoms. Schizophrenia research, 168(1-2), 537-542.

Terry, C. P. and Terry, D. L. (2015). Cell phone-related near accidents among young drivers: Associations with mindfulness. The Journal of Psychology: Interdisciplinary and Applied, 149(7), 665-683.

Thompson, B. L. and Waltz, J. (2010). Mindfulness and experiential avoidance as predictors of posttraumatic stress disorder avoidance symptom severity. Journal of Anxiety Disorders, 24(4), 409-415.

Tomfohr, L. M., Pung, M. A., Mills, P. J. and Edwards, K. (2015). Trait mindfulness is associated with blood pressure and interleukin-6: exploring interactions among subscales of the Five Facet Mindfulness Questionnaire to better understand relationships between mindfulness and health.. Journal of Behavioral Medicine, 38(1), 28-38.

Vinci, C., Spears, C. A., Peltier, M. R. and Copeland, A. L. (2016). Drinking motives mediate the relationship between facets of mindfulness and problematic alcohol use. Mindfulness, 7(3), 754-763.

Weiner, L., Wittmann, M., Bertschy, G. and Giersch, A. (2016). Dispositional mindfulness and subjective time in healthy individuals. Frontiers in Psychology, 7, 786.
