# Supplementary material for: Systematic Review and Meta-Analysis of Correlates of FFMQ Mindfulness Facets
Source: Front Psychol. 2019 Dec 6;10:2684. doi: 10.3389/fpsyg.2019.02684 (PMC6909938; doi:10.3389/fpsyg.2019.02684)
Supplement: Supplementary file 2 [file Table_2.docx]

### Table 2: Characteristics of papers included in the meta-analysis

Columns: 1: number of paper, 2: authors, 3: year, 4: open access? 5: structured abstract? 6: data available? 7: pre-registered? 8: some version of the paper freely available? 9: number of relevant studies, 10: number of samples per study, 11:number of measures; 12: sample, 13: number of participants, 14: clinical sample? 15: meditators? 16: type of sample 17: proportion of males among participants, 18: mean age, 19: standard deviation of age, 20: region in which study performed, 21: country, 22: language used in the questionnaire, 23: language of publication.

Answers to columns 14,15: y=yes, n=no, m=mixed. Type of sample: C = Community sample, I = Internet sample, S=Students, Ac=Academic, U=University community, H=Health professionals, P = other Professionals, M = Military and veterans.

| **1** | **2** | **3** | **4** | **5** | **6** | **7** | **8** | **9** | **10** | **11** |  | **12** | **13** | **14** | **15** | **16** | **17** | **18** | **19** | **20** | **21** | **22** | **23** |
| --- | --- | --- | --- | --- | --- | --- | --- | --- | --- | --- | --- | --- | --- | --- | --- | --- | --- | --- | --- | --- | --- | --- | --- |
| 1 | Adam, Heeren, Day et al. | 2015 | n | n | n | n | y | 1 | 1 | 1 |  |  | 251 | N | N | Ac | 0 | 31.81 | 11.6 | Europe | BE | FR | EN |
| 2 | Alda, Puebla-Guedea, Rodero et al. | 2016 | y | n | n | n | y | 1 | 2 | 2 |  | a | 20 | N | Y | C | 0.7 | 48.55 | 8.05 | Europe | ES | ES | EN |
|  |  |  |  |  |  |  |  |  |  |  |  | b | 20 | N | N | C | 0.7 | 48.3 | 8.76 | Europe | ES | ES | EN |
| 3 | Antonova, Amaratunga, Wright et al. | 2016 | y | n | n | n | y | 1 | 2 | 10 |  | a | 28 | N | Y | C | 1 | 39.68 | 10.19 | Europe | UK | EN | EN |
|  |  |  |  |  |  |  |  |  |  |  |  | b | 28 | N | N | C | 1 | 36.68 | 8.41 | Europe | UK | EN | EN |
| 4 | Baer, Lykins, Peters | 2012 | n | n | n | n | y | 1 | 1 | 8 |  |  | 152 | N | m | Ac | 0.3 | 44.22 | 11.8 | NAmerica | USA | EN | EN |
| 5 | Baer, Smith, Lykins et al. | 2008 | n | n | n | n | y | 1 | 4 | 4 |  | 1a | 253 | N | N | S | 0.22 | 18.9 | 3.2 | NAmerica | USA | EN | EN |
|  |  |  |  |  |  |  |  |  |  |  |  | 2a | 175 | N | N | S | 0.22 | 18.9 | 3.2 | NAmerica | USA | EN | EN |
|  |  |  |  |  |  |  |  |  |  |  |  | 1b | 267 | N | N | C | 0.4 | 49.5 | 6.7 | NAmerica | USA | EN | EN |
|  |  |  |  |  |  |  |  |  |  |  |  | 1c | 78 | N | N | Ac | 0.42 | 44.2 | 11.9 | NAmerica | USA | EN | EN |
|  |  |  |  |  |  |  |  |  |  |  |  | 2c | 75 | N | N | Ac | 0.42 | 44.2 | 11.9 | NAmerica | USA | EN | EN |
|  |  |  |  |  |  |  |  |  |  |  |  | 1d | 61 | N | Y | C | 0.32 | 48.8 | 12.9 | NAmerica | USA | EN | EN |
|  |  |  |  |  |  |  |  |  |  |  |  | 2d | 169 | N | Y | C | 0.32 | 48.8 | 12.9 | NAmerica | USA | EN | EN |
| 6 | Barnes, Lynn | 2010 | n | n | n | n | y | 1 | 1 | 3 |  |  | 102 | N | N | S | 0.32 | 18.99 | 1.9 | NAmerica | USA | EN | EN |
| 7 | Bergin, Pakenham | 2016 | n | n | n | n | n | 1 | 1 | 11 |  |  | 480 | N | N | S | 0.29 | 21.9 | 5.78 | Asia | AU | EN | EN |
| 8 | Biglan, Layton, Jones et al. | 2013 | n | n | n | n | y | 1 | 1 | 8 |  |  | 38 | N | N | P | NA | NA | NA | NAmerica | USA | EN | EN |
| 9 | Boden, Irons, Feldner et al. | 2015 | n | n | n | n | n | 1 | 1 | 6 |  |  | 979 | N | N | S | 0.23 | 19.1 | 2.2 | NAmerica | USA | EN | EN |
| 10 | Bodenlos, Wells, Noonan et al. | 2015 | n | y | n | n | y | 1 | 1 | 7 |  |  | 310 | N | N | S | 0.32 | 19.7 | 1.3 | NAmerica | USA | EN | EN |
| 11 | Bowlin, Baer | 2012 | n | n | n | n | y | 1 | 1 | 12 |  |  | 280 | N | N | S | 0.37 | 19.1 | 1.9 | NAmerica | USA | EN | EN |
| 12 | Bravo, Boothe, Pearson | 2016 | n | n | n | n | n | 1 | 1 | 13 |  |  | 688 | N | m | S | 0.33 | 22.43 | 6.99 | NAmerica | USA | EN | EN |
| 13 | Brown, Bravo, Roos et al. | 2015 | n | n | n | n | n | 1 | 1 | 7 |  |  | 944 | N | N | S | 0.35 | NA | NA | NAmerica | USA | EN | EN |
| 14 | Caluyong, Zambrana, Romanow et al. | 2015 | n | n | n | n | y | 1 | 1 | 9 |  |  | 74 | Y | N | C | 0.61 | 63.4 | 10.2 | NAmerica | CA | NA | EN |
| 15 | Camilleri, Mejean, Bellisle et al. | 2015 | y | y | n | y | y | 1 | 1 | 2 |  | a | 49228 | N | N | C | 0 | 47.1 | 14.2 | Europe | FR | FR | EN |
|  |  |  |  |  |  |  |  |  |  |  |  | b | 14400 | N | N | C | 1 | 53.6 | 14.4 | Europe | FR | FR | EN |
| 16 | Campos, Botella, Quero et al. | 2015 | n | n | n | n | y | 1 | 1 | 2 |  |  | 365 | N | m | C | 0.32 | 41.87 | 11.18 | Europe | ES | ES | EN |
| 17 | Cebolla, Garcia-Palacios, Soler et al. | 2012 | y | y | n | n | y | 1 | 1 | 9 |  |  | 462 | m | N | S | 0.5 | 27.4 | 8.3 | Europe | ES | ES | EN |
| 18 | Christopher, Neuser, Michael et al. | 2012 | n | n | n | n | n | 1 | 1 | 6 |  |  | 349 | N | m | I | 0.25 | 32.44 | 11.73 | NAmerica | USA | EN | EN |
| 19 | Consedine, Butler | 2014 | n | n | n | n | y | 1 | 1 | 10 |  |  | 121 | N | N | C | 0.33 | NA | NA | Asia | NZ | EN | EN |
| 20 | Corthorn, Milicic | 2016 | n | n | n | n | y | 1 | 1 | 7 |  |  | 62 | N | N | U | 0 | 36 | 5.1 | LatAmerica | CL | ES | EN |
| 21 | Curtiss, Klemanski | 2014 | n | n | n | n | n | 1 | 1 | 11 |  |  | 151 | Y | N | C | 0.36 | 38 | 14.8 | NAmerica | USA | EN | EN |
| 22 | Curtiss, Klemanskia | 2014 | n | y | n | n | n | 1 | 1 | 3 |  |  | 151 | Y | N | C | 0.36 | 38 | 14.8 | NAmerica | USA | EN | EN |
| 23 | Day, Smitherman, Ward et al. | 2015 | n | y | n | n | y | 1 | 1 | 2 |  |  | 214 | N | N | S | 0.11 | 18.7 | 2.3 | NAmerica | USA | EN | EN |
| 24 | Desrosiers, Klemanski, Nolen-Hoeksema | 2013 | n | n | n | n | y | 1 | 1 | 4 |  |  | 187 | Y | N | C | 0.35 | 38 | 14.2 | NAmerica | USA | EN | EN |
| 25 | Elices, Pascual, Carmona et al. | 2015 | y | n | n | n | y | 1 | 1 | 10 |  |  | 100 | Y | N | C | 0.12 | 30.46 | 6.84 | Europe | ES | ES | EN |
| 26 | Emanuel, Updegraff, Kalmbach et al. | 2010 | n | n | n | n | y | 1 | 1 | 2 |  |  | 220 | N | N | S | 0.22 | 23.3 | NA | NAmerica | USA | EN | EN |
| 27 | Evans, Baer, Segerstrom | 2009 | n | n | n | n | n | 1 | 1 | 5 |  |  | 142 | N | N | S | 0.28 | 19.33 | 2.72 | NAmerica | USA | EN | EN |
| 28 | Evans, Segerstrom | 2011 | n | n | n | n | y | 1 | 1 | 3 |  |  | 199 | N | N | S | NA | NA | NA | NAmerica | USA | EN | EN |
| 29 | Fatter, Hayes | 2013 | n | n | n | n | y | 1 | 1 | 6 |  |  | 100 | N | m | H | 0.31 | NA | NA | NAmerica | USA | EN | EN |
| 30 | Fernandez, Wood, Stein et al. | 2010 | n | n | n | n | y | 1 | 1 | 4 |  |  | 316 | N | N | S | 0.44 | 22 | 0.41 | NAmerica | USA | EN | EN |
| 31 | Fink, Foran, Sweeney et al. | 2009 | n | n | n | n | n | 1 | 1 | 3 |  |  | 79 | N | N | S | 0 | 21.61 | NA | NAmerica | USA | EN | EN |
| 32 | Frank, Nara, Zavagnin et al. | 2015 | n | n | n | n | y | 1 | 1 | 2 |  |  | 76 | N | N | C | NA | 45.6 | 25 | NAmerica | USA | EN | EN |
| 33 | Fuller, Sainsbury, Caterson et al. | 2017 | n | y | n | y | n | 1 | 1 | 2 |  |  | 137 | Y | N | C | 0.44 | 59.8 | 10.4 | Asia | AU | EN | EN |
| 34 | Garland, Boettiger, Gaylord et al. | 2012 | n | n | n | n | y | 1 | 1 | 7 |  |  | 58 | Y | N | C | 0.81 | 39.8 | 9.3 | NAmerica | USA | EN | EN |
| 35 | Garland, Campbell, Samuels et al. | 2013 | n | n | n | n | y | 1 | 1 | 5 |  |  | 111 | Y | N | C | 0.28 | 58.89 | 11.18 | NAmerica | CA | EN | EN |
| 36 | Garland, Roberts-Lewis | 2013 | n | n | n | n | y | 1 | 1 | 4 |  |  | 125 | Y | N | C | 0.92 | 38.7 | 10 | NAmerica | USA | EN | EN |
| 37 | Gonzalez, Locicero, Mahaffey et al. | 2016 | n | n | n | n | y | 1 | 1 | 5 |  |  | 137 | Y | N | C | 0.85 | 48.94 | 8.89 | NAmerica | USA | EN | EN |
| 38 | Goodall, Trejnowska, Darling | 2012 | n | n | n | n | y | 1 | 1 | 8 |  |  | 194 | N | N | I | 0.16 | 26.6 | NA | Europe | UK | EN | EN |
| 39 | Gorbovskaya, Park, Kim | 2014 | n | n | n | n | y | 1 | 1 | 3 |  |  | 54 | N | N | S | 0.52 | 22.63 | 3.48 | NAmerica | CA | EN | EN |
| 40 | Gu, Strauss, Crane et al. | 2016 | n | n | n | n | y | 1 | 1 | 4 |  |  | 238 | Y | N | C | 0.29 | 49.18 | 12.01 | Europe | UK | EN | EN |
| 41 | Hamill, Pickett, Amsbaugh et al. | 2015 | n | n | n | n | n | 1 | 1 | 9 |  |  | 467 | N | N | S | 0.23 | 20.99 | 4.92 | NAmerica | USA | EN | EN |
| 42 | Hanley, Garland | 2014 | n | n | n | n | y | 1 | 5 | 1 |  | 1 | 329 | N | N | I | 0.31 | 35 | 12.89 | NAmerica | USA | EN | EN |
|  |  |  |  |  |  |  |  |  |  |  |  | 2 | 130 | N | Y | I | 0.25 | 38 | 13.14 | NAmerica | USA | EN | EN |
|  |  |  |  |  |  |  |  |  |  |  |  | 3 | 101 | N | N | S | 0.27 | 22 | 5.64 | NAmerica | USA | EN | EN |
|  |  |  |  |  |  |  |  |  |  |  |  | 4 | 115 | N | Y | C | 0.32 | 48 | 14 | NAmerica | USA | EN | EN |
|  |  |  |  |  |  |  |  |  |  |  |  | 5 | 58 | N | Y | C | 0.81 | 40 | 9.3 | NAmerica | USA | EN | EN |
| 43 | Hanley, Palejwala, Hanley et al. | 2015 | n | n | n | n | y | 1 | 1 | 6 |  |  | 243 | N | N | S | 0.15 | 20 | 4.19 | NAmerica | USA | EN | EN |
| 44 | Heeren, Douilliez, Peschard et al.a | 2011 | n | n | n | n | y | 1 | 1 | 2 |  |  | 214 | N | N | Ac | 0.38 | 35.39 | 13.55 | Europe | BE,FR | FR | EN |
| 45 | Hollis-Walker, Colosimo | 2011 | n | n | n | n | n | 1 | 1 | 2 |  |  | 123 | N | N | S | 0.22 | 20.9 | NA | NAmerica | USA | EN | EN |
| 46 | Hutchison, Gunthert | 2013 | n | n | n | n | n | 1 | 1 | 7 |  |  | 316 | N | N | S | 0.23 | NA | NA | NAmerica | USA | EN | EN |
| 47 | Jennings | 2015 | n | n | n | n | y | 1 | 1 | 5 |  |  | 35 | N | N | P | 0.09 | 45.52 | 12.1 | NAmerica | USA | EN | EN |
| 48 | Johns, Allen, Gordon | 2015 | n | n | n | n | n | 1 | 1 | 5 |  |  | 94 | N | N | I | 0.42 | 42 | 12.56 | NAmerica | USA | EN | EN |
| 49 | Jones, Hansen | 2015 | n | n | n | n | y | 1 | 1 | 5 |  |  | 215 | N | N | S | NA | 21.3 | 3.42 | NAmerica | USA | EN | EN |
| 50 | Jones, Mist, Casselberry et al. | 2015 | n | y | n | n | y | 1 | 1 | 1 |  |  | 4986 | Y | N | I | 0.03 | 52.2 | 10.6 | Global | Global | EN | EN |
| 51 | Kalill, Treanor, Roemer | 2014 | n | n | n | n | y | 1 | 1 | 5 |  |  | 157 | N | N | S | 0.36 | NA | NA | NAmerica | USA | EN | EN |
| 52 | Karyadi, Cyders | 2015 | n | n | n | n | y | 1 | 1 | 4 |  |  | 240 | N | N | S | 0.3 | 19.37 | 1.65 | NAmerica | USA | EN | EN |
| 53 | Keane | 2014 | n | n | n | n | n | 1 | 1 | 4 |  |  | 40 | N | Y | H | 0.38 | NA | NA | Europe | IRE | EN | EN |
| 54 | Kearney, Malte, Mc, Manus et al. | 2013 | n | n | n | y | y | 1 | 1 | 5 |  |  | 42 | Y | N | M | 0.6 | 53.6 | 8.6 | NAmerica | USA | EN | EN |
| 55 | Khaddouma, Gordon, Bolden | 2015 | n | n | n | n | y | 1 | 1 | 2 |  |  | 322 | N | N | S | 0.24 | 18.79 | 2.35 | NAmerica | USA | EN | EN |
| 56 | Kraemer, Mc, Leish, Johnson | 2015 | n | n | n | n | y | 1 | 1 | 5 |  |  | 56 | Y | N | S | 0.3 | 19.5 | 2.7 | NAmerica | USA | EN | EN |
| 57 | Lara, Herrero, Blanco-Donoso et al. | 2015 | n | n | n | n | y | 1 | 1 | 5 |  |  | 1210 | N | N | S | 0.32 | 20.12 | 5.15 | LatAmerica | MX | ES | ES |
| 58 | Lattimore, Fisher, Malinowski | 2011 | n | n | n | n | y | 1 | 1 | 9 |  |  | 190 | N | N | C | 0 | 26 | 0.6 | NAmerica | USA | EN | EN |
| 59 | Lavender, Gratz, Tull | 2011 | n | n | n | n | n | 1 | 1 | 9 |  |  | 276 | N | N | S | 0 | 20.3 | 2.6 | NAmerica | USA | EN | EN |
| 60 | Lee, Harvey, Price et al. | 2016 | n | y | n | n | n | 1 | 1 | 8 |  |  | 80 | Y | N | C | 0.24 | 60.3 | 10.3 | NAmerica | USA | EN | EN |
| 61 | Levin, Dalrymple, Himes et al. | 2014 | n | n | n | n | y | 1 | 1 | 6 |  |  | 820 | Y | N | C | 0.19 | 42.93 | 11.4 | NAmerica | USA | EN | EN |
| 62 | Levin, Dalrymple, Zimmerman | 2014 | n | n | n | n | y | 1 | 1 | 3 |  |  | 867 | Y | N | C | 0.44 | 39.29 | 14.24 | NAmerica | USA | EN | EN |
| 63 | Lustyk, Gerrish, Douglas et al. | 2011 | n | n | n | n | y | 1 | 1 | 10 |  |  | 127 | N | N | S | 0 | NA | NA | NAmerica | USA | EN | EN |
| 64 | Michalak, Zarbock, Drews et al. | 2016 | n | n | n | n | n | 1 | 1 | 8 |  |  | 550 | N | N | S | 0.19 | 24.4 | 6.17 | Europe | DE | DE | DE |
| 65 | Mira, Campos, Etchemendy et al. | 2016 | n | n | n | n | y | 1 | 1 | 2 |  |  | 60 | N | N | S | 0.23 | 22.9 | 3.85 | Europe | ES | ES | EN |
| 66 | Montgomery, Norman, Messenger et al. | 2016 | n | y | n | n | n | 1 | 1 | 5 |  |  | 120 | Y | N | C | 0.29 | 45.92 | 18.41 | Europe | UK | EN | EN |
| 67 | Murphy, Mac, Killop | 2012 | n | y | n | n | y | 1 | 1 | 8 |  |  | 116 | N | N | S | 0.2 | 20.3 | 1.3 | NAmerica | USA | EN | EN |
| 68 | Neale-Lorello, Haaga | 2015 | n | n | n | n | y | 1 | 2 | 2 |  | a | 100 | N | Y | C | 0.72 | 41.81 | 13.11 | NAmerica | USA | EN | EN |
|  |  |  |  |  |  |  |  |  |  |  |  | b | 90 | N | N | S | 0.76 | 27.73 | 10.39 | NAmerica | USA | EN | EN |
| 69 | Ostafin, Brooks, Laitem | 2014 | n | n | n | n | y | 2 | 1 | 2 |  | 1 | 80 | N | N | S | 0.53 | 20.05 | 2.43 | NAmerica | USA | EN | EN |
|  |  |  |  |  |  |  |  |  |  |  |  | 2 | 68 | N | N | S | 0.59 | 19.68 | 3.32 | NAmerica | USA | EN | EN |
| 70 | Pearson, Lawless, Brown et al. | 2015 | n | n | n | n | y | 1 | 1 | 1 |  |  | 941 | N | N | S | 0.36 | 20.55 | 4.35 | NAmerica | USA | EN | EN |
| 71 | Pepping, ODonovan, Davis | 2013 | n | n | n | n | y | 1 | 1 | 2 |  |  | 329 | N | N | S | 0.27 | 21.53 | 6.59 | NAmerica | USA | EN | EN |
| 72 | Pepping, ODonovan, Davis | 2014 | n | n | n | n | y | 1 | 2 | 2 |  | a | 225 | N | N | S | 0.28 | 21.06 | 6.08 | NAmerica | USA | EN | EN |
|  |  |  |  |  |  |  |  |  |  |  |  | b | 65 | N | N | S | 0.23 | 23.78 | 8.82 | NAmerica | USA | EN | EN |
| 73 | Peters, Eisenlohr-Moul, Upton et al. | 2013 | n | n | n | n | y | 1 | 1 | 6 |  |  | 227 | N | N | S | 0.33 | 19.01 | 1.11 | NAmerica | USA | EN | EN |
| 74 | Peters, Erisman, Upton et al. | 2011 | n | n | n | n | y | 2 | 1 | 11 |  | 1 | 347 | N | N | U | 0.4 | 23.73 | 7.66 | NAmerica | USA | EN | EN |
|  |  |  |  |  |  |  |  |  |  |  |  | 2 | 227 | N | N | S | 0.33 | 19.01 | 1.11 | NAmerica | USA | EN | EN |
| 75 | Peters, Smart, Eisenlohr-Moul et al. | 2015 | n | n | n | n | y | 1 | 1 | 5 |  |  | 823 | N | N | S | 0.29 | 19.25 | 2.51 | NAmerica | USA | EN | EN |
| 76 | Quezada-Berumen, Gonzalez-Ramirez, Cebolla et al. | 2014 | n | n | n | n | y | 1 | 1 | 2 |  |  | 578 | N | m | I | 0.38 | 41.3 | 11.2 | Europe | ES | ES | ES |
| 77 | Raphiphatthana, Jose, Kielpikowski | 2016 | n | n | n | n | y | 2 | 1 | 3 |  | 1 | 284 | N | N | S | 0.31 | NA | NA | Asia | NZ | EN | EN |
|  |  |  |  |  |  |  |  |  |  |  |  | 2 | 228 | N | N | S | 0.25 | NA | NA | Asia | NZ | EN | EN |
| 78 | Reese, Zielinski, Veilleux | 2015 | n | n | n | n | y | 1 | 1 | 7 |  |  | 242 | N | N | S | 0.38 | 19.28 | 2.06 | NAmerica | USA | EN | EN |
| 79 | Rice, Boykin, Jeter et al. | 2013 | n | n | n | n | n | 1 | 1 | 1 |  |  | 29 | N | N | M | 0.57 | 45.3 | 11.97 | NAmerica | USA | EN | EN |
| 80 | Roos, Pearson, Brown | 2015 | n | n | n | n | y | 1 | 1 | 8 |  |  | 297 | N | N | S | 0.35 | 21.54 | 5.77 | NAmerica | USA | EN | EN |
| 81 | Royuela-Colomer, Calvete | 2016 | n | n | n | n | n | 1 | 1 | 3 |  |  | 520 | N | N | S | 0.42 | 16.11 | 0.98 | Europe | ES | ES | EN |
| 82 | Ruiz, Odriozola-Gonzalez, Suarez-Falcon | 2014 | y | n | n | n | y | 1 | 1 | 3 |  |  | 105 | N | N | S | 0.26 | 23.17 | 4.41 | Europe | ES | ES | EN |
| 83 | Schmidt, Vinet | 2015 | y | n | n | n | y | 1 | 1 | 11 |  |  | 399 | N | N | S | 0.59 | 19.9 | 1.97 | LatAmerica | CL | ES | ES |
| 84 | Seli, Carriere, Smilek | 2015 | n | n | n | n | y | 1 | 1 | 3 |  | a | 716 | N | N | S | NA | NA | NA | NAmerica | CA | EN | EN |
|  |  |  |  |  |  |  |  |  |  |  |  | b | 762 | N | N | S | NA | NA | NA | NAmerica | CA | EN | EN |
| 85 | Shorey, Seavey, Quinn et al. | 2014 | n | y | n | n | y | 1 | 1 | 7 |  |  | 481 | N | N | S | 0 | 18.58 | 1.28 | NAmerica | USA | EN | EN |
| 86 | Short, Mazmanian | 2013 | n | n | n | n | n | 1 | 1 | 10 |  |  | 213 | N | N | S | 0.17 | 25 | 7.4 | NAmerica | CA | EN | EN |
| 87 | Short, Mazmanian, Oinonen et al. | 2016 | n | n | n | n | y | 1 | 1 | 12 |  |  | 77 | N | N | S | 0.29 | 21.23 | 5.98 | NAmerica | CA | EN | EN |
| 88 | Slonim, Kienhuis, Di, Benedetto et al. | 2015 | n | y | n | n | y | 1 | 1 | 11 |  |  | 207 | N | N | S | 0.33 | 21.82 | 3.62 | Asia | AU | EN | EN |
| 89 | Soler, Franquesa, Feliu-Soler et al. | 2014 | n | n | n | n | y | 1 | 1 | 1 |  |  | 921 | m | N | C | 0.33 | 38.3 | 11.9 | Europe | ES | ES | EN |
| 90 | Soler, Soriano, Ferraz et al. | 2013 | n | n | n | n | n | 1 | 1 | 1 |  |  | 57 | Y | N | C | 0.04 | 29.4 | 8.9 | Europe | ES | ES | EN |
| 91 | Spofford, Nevels, Gontkovsky et al. | 2014 | n | n | n | n | n | 1 | 1 | 2 |  |  | 164 | N | N | S | 0.34 | NA | NA | NAmerica | USA | EN | EN |
| 92 | Tabak, Horan, Green | 2015 | n | n | n | n | n | 1 | 1 | 6 |  | a | 35 | Y | N | C | 0.54 | 46.83 | 9.93 | NAmerica | USA | EN | EN |
|  |  |  |  |  |  |  |  |  |  |  |  | b | 25 | N | N | I | 0.52 | 46.6 | 8.32 | NAmerica | USA | EN | EN |
| 93 | Terry, Terry | 2015 | n | n | n | n | n | 1 | 1 | 6 |  |  | 385 | N | N | S | 0.52 | 19 | 1.2 | NAmerica | USA | EN | EN |
| 94 | Thompson, Waltz | 2010 | n | n | n | n | n | 1 | 1 | 8 |  |  | 191 | Y | N | S | NA | NA | NA | NAmerica | CA | EN | EN |
| 95 | Tomfohr, Pung, Mills et al. | 2015 | n | n | n | n | y | 1 | 1 | 5 |  |  | 130 | N | N | C | 0.44 | 21.7 | 2.7 | NAmerica | USA | EN | EN |
| 96 | Vinci, Spears, Peltier et al. | 2016 | n | n | n | n | y | 1 | 1 | 5 |  |  | 207 | Y | N | S | 0.24 | 20.13 | 1.89 | NAmerica | USA | EN | EN |
| 97 | Weiner, Wittmann, Bertschy et al. | 2016 | y | n | n | n | y | 1 | 1 | 8 |  |  | 117 | N | N | Ac | 0.41 | 26 | 7.7 | Europe | FR | FR | EN |
